# Data‐Driven Modeling of Composition–Processing–Microstructure Relations for Recycled Aluminum Cast Alloys

**DOI:** 10.1002/advs.75446

**Published:** 2026-04-30

**Authors:** Jaemin Wang, Waleed Mohammed, Dierk Raabe

**Affiliations:** ^1^ Max Planck Institute For Sustainable Materials Düsseldorf Germany; ^2^ Department of Materials Science and Metallurgical Engineering Kyungpook National University Daegu Republic of Korea

**Keywords:** aluminum recycling, composition–process–structure linkage, iron impurity, machine learning, sustainable alloys

## Abstract

Aluminum is the second most widely used metal worldwide, with essential roles in transportation, construction, packaging, and energy infrastructure. Recycling aluminum can save up to 95% of the energy required for primary production, making it a cornerstone of low‐carbon manufacturing. However, recycled aluminum alloys inevitably contain higher levels of iron, which promotes the formation of brittle microscopic phases that degrade performance and limit industrial use. These phases can appear in different forms and dispersion depending on alloy composition and processing. Yet, practical guidelines for controlling them remain largely empirical and qualitative, especially when data are limited. To improve this situation, this work separates two key questions that are often conflated in alloy design: which phase type forms, and how strongly that phase affects material behavior once it appears. Using data representative of industrial conditions, we show how alloy chemistry and processing history determine which phases form and govern whether these phases develop as a few large, platelet‐like features or as many small, compact particles. By clarifying these distinct roles of composition and processing, the results provide a simple and actionable framework for mitigating deleterious iron‐rich phases in recycled aluminum alloys and enabling more quantitative, predictable, and impurity‐tolerant sustainable alloy design.

## Graphical abstract

Interpretable machine learning reveals how composition and processing govern the formation and microstructural burden of Fe‐rich intermetallic compounds in recycled Al–Si–Fe–Mn alloys. By separating morphology selection from morphology‐conditioned burden partitioning, this framework shows that identical Fe contents can yield different intermetallic architectures. The results provide actionable design pathways for mitigating deleterious Fe‐intermetallic microstructures under recycling‐driven composition variability.

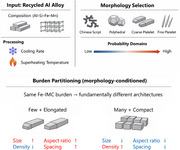

## 1 Introduction

**FIGURE 1:**
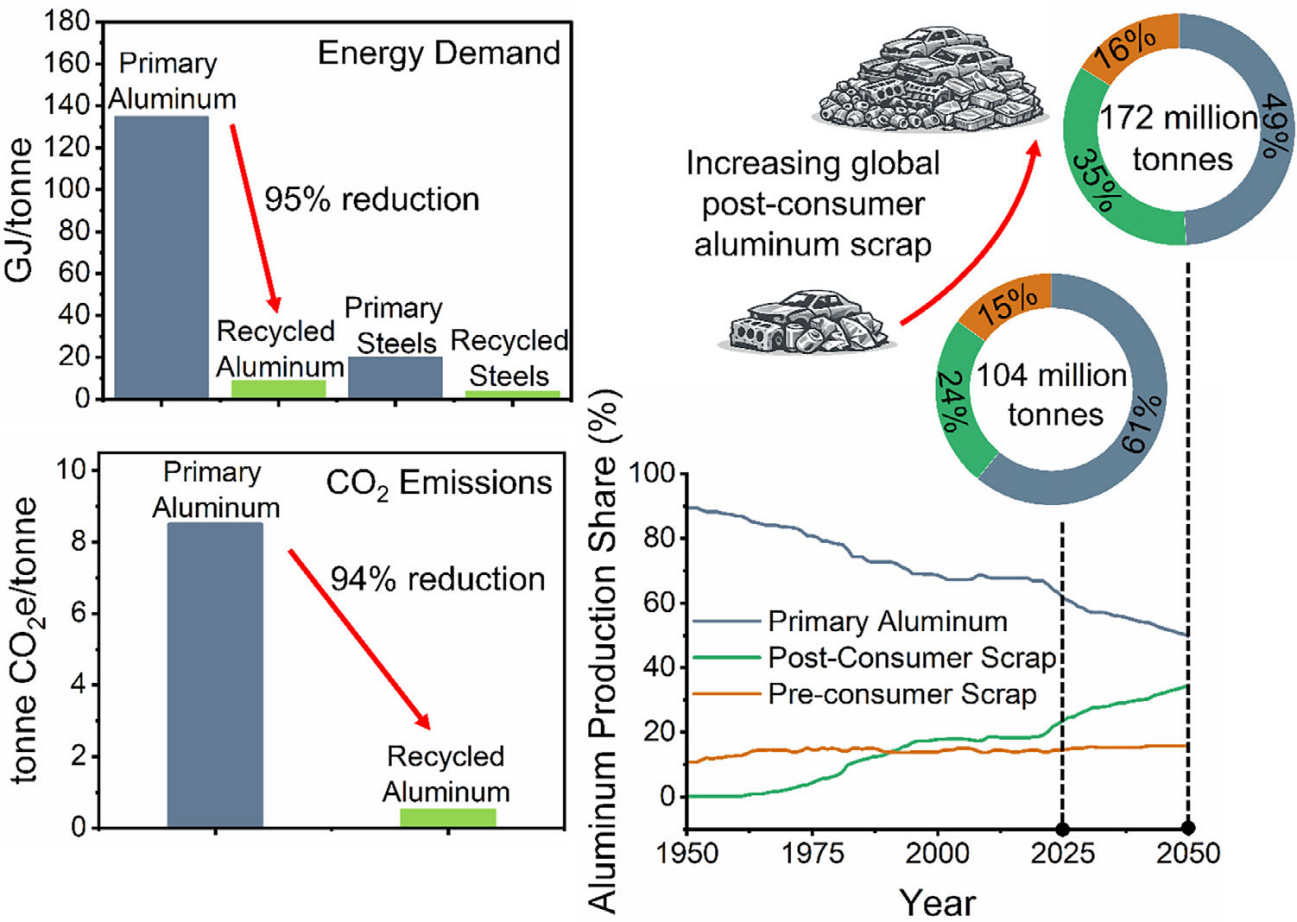
Global context and sustainability motivation for the use of high‐performance recycled aluminum alloys [5, 6]. Aluminum recycling through scrap remelting requires 95% less energy than primary production from ores and produces 94% less greenhouse gas emissions. Global aluminum production exceeded 100 million tons in 2025, of which 24% of the aluminum produced came from end‐of‐life products like used beverage cans, trashed packaging foils, and scrapped vehicles. The amount of post‐consumer scrap available for recycling is expected to rise sharply. Data were obtained from the International Aluminum Institute [6].

Aluminum underpins modern transportation, buildings, and electrification, and its global production has reached the scale of more than one hundred million tons per year [1]. At the same time, primary aluminum production remains emissions‐intensive, mainly because of the high carbon footprint of the grid electricity demand for electrolytic reduction, contributing on the order of one gigaton of CO_2_‐equivalent emissions annually and accounting for nearly 3% of global greenhouse gas emissions [1, 2, 3, 4]. Recycling therefore represents one of the most immediately scalable levers for decarbonizing metal supply chains, as remelting scrap requires only a small fraction of the energy associated with primary production [1, 2]. These sustainability drivers are expected to accelerate the use of secondary aluminum in high‐volume applications, particularly in the automotive sector [1, 2]. Figure 1 provides a quantitative overview of the global aluminum–scrap ecosystem and the sustainability motivation for improving recycled‐alloy performance.

**FIGURE 2:**
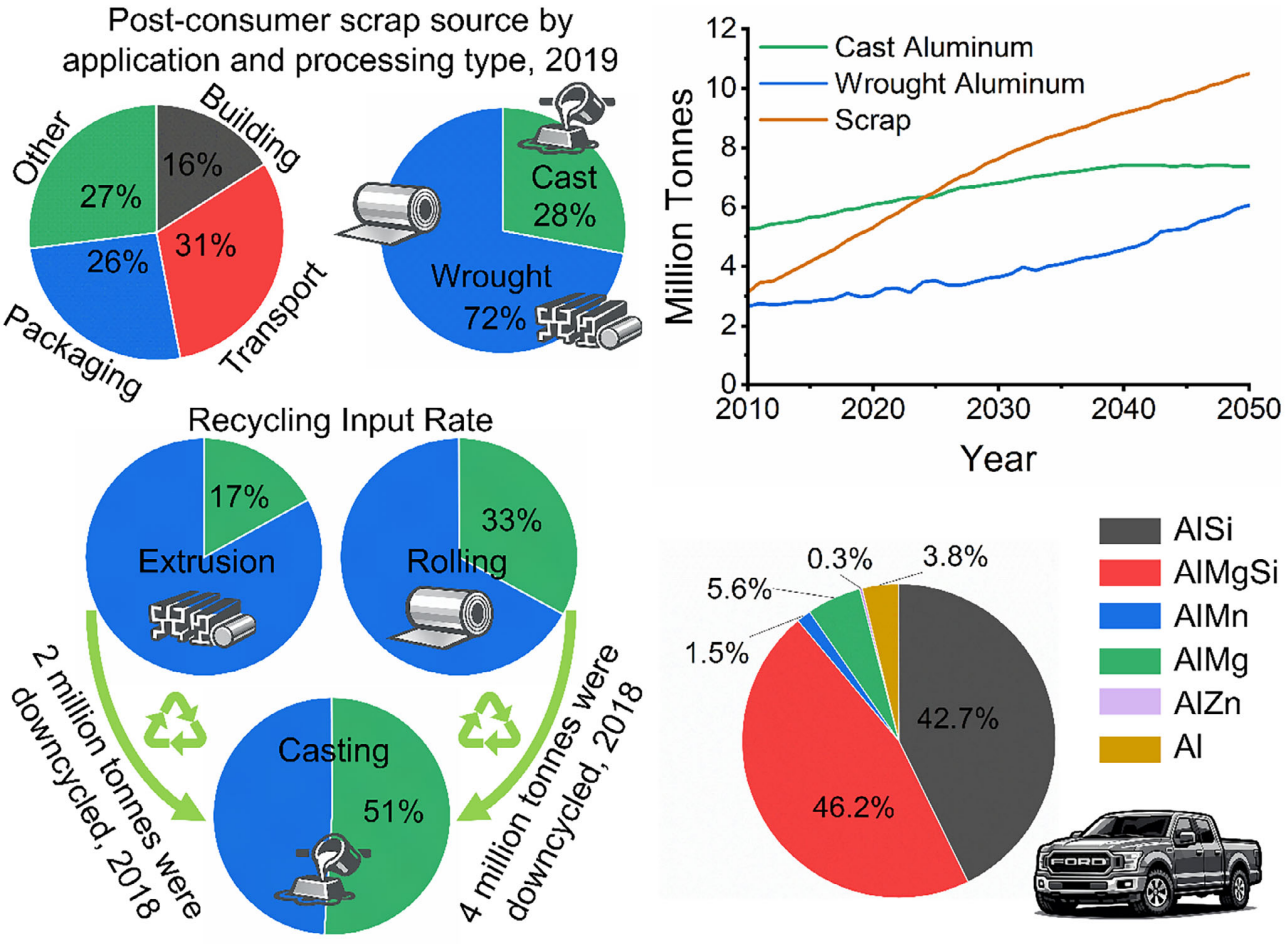
The central role of cast Al–Si alloys as a sink for post‐consumer aluminum scrap. (a) In 2019, approximately 28% of post‐consumer aluminum scrap originated from cast alloys, which are primarily used in internal combustion engine vehicles [12]. (b) Cast Al–Si alloys constitute a significant share of the aluminum content in internal combustion engine vehicles, as illustrated in the example of a typical pickup truck such as the Ford F‐150 [10]. (c) Across all end‐use sectors, cast aluminum alloys exhibit the highest recycling input rate (i.e., highest recycled content); in 2018, approximately 6 million tons of wrought (rolled and extruded) aluminum were downcycled and ended as part of cast products [6]. (d) However, the growing demand for electric vehicles is expected to reduce the use of cast aluminum in the transportation sector, since electric vehicles rely more heavily on wrought (rolled and extruded) aluminum alloys than cast ones. If cast Al–Si alloys are not upcycled into value‐added products, the amount of discarded aluminum scrap from end‐of‐life vehicles may eventually exceed the demand for automotive cast aluminum alloys [13]. This would shift the transportation sector from functioning as a scrap sink to becoming a net scrap source. This broader context motivates the need for quantitative, microstructure‐aware design strategies for recycling and upcycling Al–Si alloys.

Cast Al–Si alloys are among the most widely used aluminum alloy families, owing to their excellent castability, favorable strength‐to‐weight ratio, high thermal conductivity, and cost efficiency [7]. Importantly, they also serve as a major “sink” for aluminum scrap, because cast alloys can accept higher impurity levels than many wrought products [1, 8, 9]. The high impurity tolerance of Al–Si cast alloys makes them premier candidate materials for upcycling post‐consumer scrap. By accommodating the chemical fluctuations inherent in low‐cost secondary scrap streams, these alloys can transform less predictable, compositionally variable feedstock into high‐value engineering materials [1, 8, 9]. However, the landscape of global scrap streams is currently undergoing a significant shift: particularly the ongoing transition from internal combustion engine vehicles to electric vehicles is expected to reshape both the global demand setting and the volume and composition of the scrap return flows of Al–Si components [10, 11]. Cast Al–Si alloys are the dominant aluminum alloy family used in internal combustion engine vehicle components, including engine blocks, pistons, cylinder heads, crankcase covers, and transmission and drivetrain housings [10, 11]. These cast Al‐Si components can account for up to 65% of the total aluminum content per average European vehicle [10, 11]. As large volumes of Al–Si scrap enter the recycling stream, a central question becomes how to upcycle these alloys into higher‐value products without losing their role as an impurity sink [10, 11]. Figure 2 highlights the pivotal role of cast Al–Si alloys as a key sink for post‐consumer scrap, as well as the projected influence of increasing electric vehicle demand on the recycling system.

**FIGURE 3:**
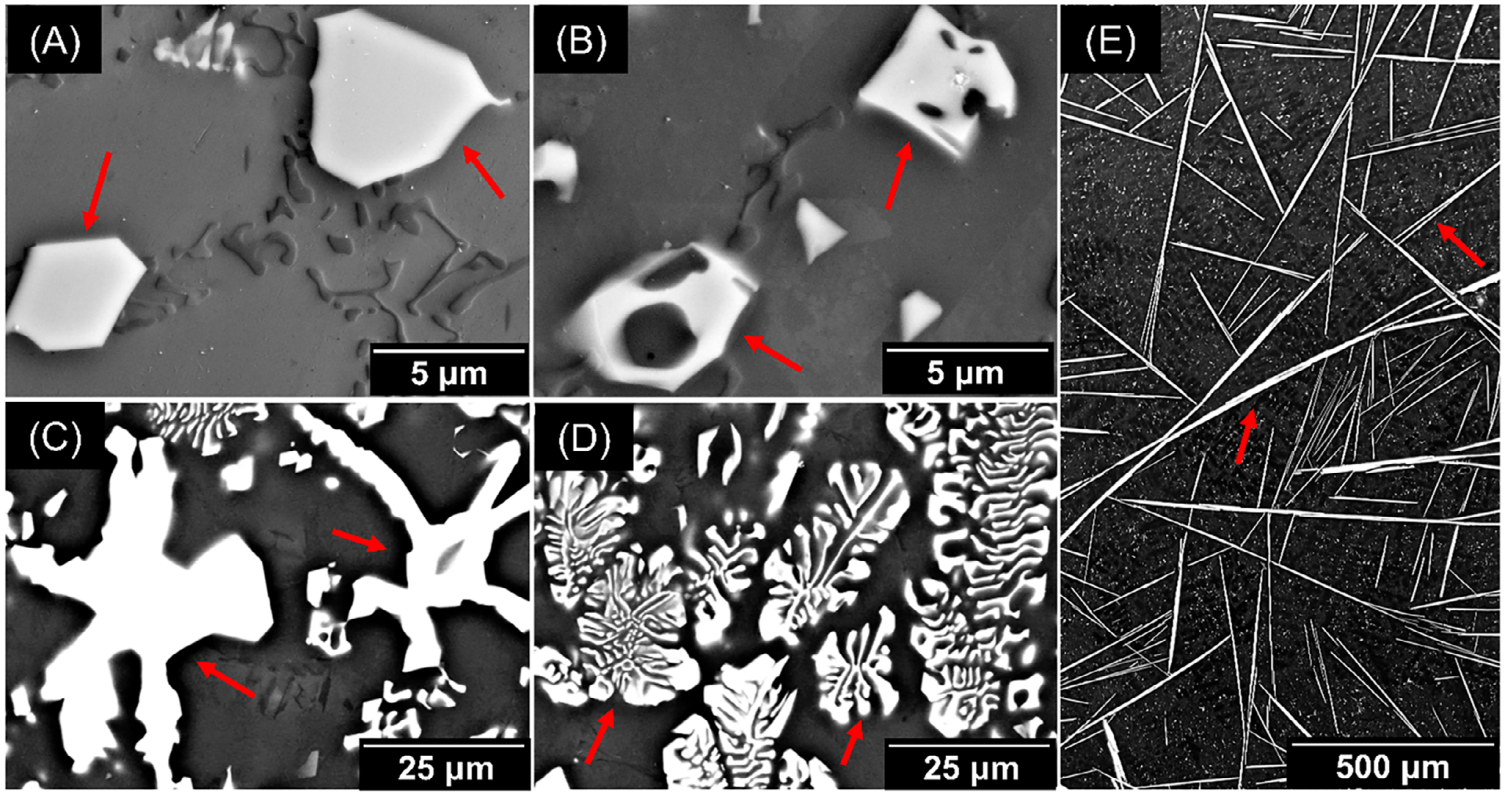
Morphological forms of Fe‐rich intermetallic compounds in cast Al–Si–Fe–Mn alloys. (A) Solid polyhedral. (B) Hollowed polyhedral. (C) Branched star‐like polyhedral. (D) Chinese‐script. (E) Platelet. Platelet‐like Fe‐IMCs act as strong stress concentrators and are particularly detrimental to ductility, whereas compact polyhedral and Chinese‐script morphologies distribute the same chemical burden in a mechanically less harmful manner. This contrast underscores why morphology, along with size and spatial arrangement, are critical descriptors for assessing the severity of Fe contamination in recycled aluminum alloys, rather than phase fraction alone.

The main barrier to the recycling and upcycling of scrap cast Al‐Si alloys is the accumulation of impurity elements that deteriorate properties and complicate downstream processing. Among them, iron is widely recognized as the most critical, because its solid solubility in aluminum is extremely low and even small concentrations promote the precipitation of hard, brittle iron‐rich particles (often called Fe‐containing intermetallic compounds, Fe‐IMCs) [1, 14, 15, 16]. From a microstructural perspective, the central challenge is therefore not merely the presence of Fe‐IMCs, but the morphological form in which iron is accommodated. Platelet‐like Fe‐IMCs act as sharp stress concentrators and facilitate crack initiation and propagation, whereas more compact morphologies, such as polyhedral and Chinese‐script‐like, distribute the same chemical burden in a mechanically less harmful way. This distinction motivates us to place here explicit focus on the micromechanically highly relevant phase morphology, not only on phase fraction. Figure 3 illustrates representative Fe‐IMC morphologies and why morphology governs damage sensitivity, and Table 1 summarizes the most common Fe‐IMC types reported in cast Al–Si–Fe–Mn alloys together with their typical morphological signatures [1, 14, 15, 16, 17].

**TABLE 1:** Overview of representative Fe‐rich intermetallic compounds in cast Al–Si–Fe–Mn alloys.

Phase (nominal)	Typical morphology	Mechanical impact
β‐Al_9_Fe_2_Si_2_	Platelet	Strong stress concentrator, severely degrades ductility
δ‐Al_4_FeSi_2_		
α‐Al_15_(Fe,Mn)_3_Si_2_	Polyhedral (solid, hollow, or branched star‐like) / Chinese script	Relatively benign compared to platelets, load more evenly distributed
α‐Al_8_Fe_2_Si		
γ‐Al_3_FeSi		

In practice, industry seeks to suppress harmful platelet‐like Fe‐IMCs while promoting polyhedral or Chinese‐script morphologies by adjusting chemistry (especially Si, Fe, and Mn) and by tuning solidification conditions such as cooling rate and melt superheating [1, 14, 15, 16, 17]. However, multiple experimental studies have shown that these effects are strongly non‐linear and coupled: changes in Fe, Mn, Si, cooling rate, and melt thermal history can shift not only the amount of Fe‐IMCs formed, but also the dominant morphology and the associated size, spacing, and connectivity [17, 18, 19, 20, 21, 22]. Seemingly similar alloy compositions can yield different outcomes under small variations in processing, and conversely, similar morphologies can arise through different chemistry–processing pathways [17, 23, 24]. These observations underscore why existing design guidelines remain largely empirical and why it remains difficult to make robust, quantitative predictions of Fe‐IMC morphology and severity under industrially accessible casting conditions.

Physics‐based tools also face limitations in this field. Thermodynamic equilibrium calculations and classical non‐equilibrium solidification approximations often deviate from experimentally observed Fe‐IMC behavior under far‐from‐equilibrium casting conditions and under the complex interaction of multiple transition elements [17, 23, 24]. Moreover, it should be noted that complex engineering alloys are typically in a transient thermodynamic state rather than in thermodynamic equilibrium. Accordingly, thermodynamic equilibrium considerations define the bounds of the system, whereas the actual microstructure evolution is governed by the imposed manufacturing conditions and their translation into microstructural kinetics. Consequently, thermodynamic descriptions alone are insufficient to predict microstructure formation, as the morphology, size, and spatial distribution of intermetallic phases are strongly influenced by kinetic factors such as diffusion, interface mobility, and solidification pathways. In principle, these effects can be addressed by coupling thermodynamic calculations with kinetic simulations (e.g., phase‐field or solidification modeling). However, such approaches require reliable atomic mobility databases and kinetic parameters, which are often incomplete or unavailable for multicomponent Al–Si–Fe–Mn systems under industrially relevant conditions.

As a result, a quantitative composition–process–microstructure linkage that is reliable under realistic industrial variability is still lacking. Such a linkage is increasingly important, not only for alloy/process selection, but also for constructing statistically representative microstructures for fracture simulations and property prediction, and for enabling inverse design and optimization workflows without requiring extensive human‐in‐the‐loop iteration.

Machine learning offers an attractive complementary route for navigating this coupled design space with a limited experimental budget. Here, machine learning is not used as a black‐box predictor, but as a structured surrogate for complex solidification behavior under industrially relevant, far‐from‐equilibrium conditions. The emphasis is therefore on interpretability and regime identification, revealing which levers matter and where transitions occur, rather than on high‐precision numerical prediction. This is particularly important for microstructural descriptors that are sparse or subject to measurement uncertainty, where overly confident regression models can be misleading.

The present study addresses three linked questions that also reflect how metallurgists reason about Fe‐IMCs. First, given alloy chemistry and processing conditions, which morphology type of Fe‐IMC is most likely to form? Second, why does that morphology emerge, meaning which chemistry–processing couplings dominate the transition? Third, once a morphology forms, how severe is it, namely how the intermetallic burden is partitioned into area fraction, feature density, characteristic size, shape, and spacing regimes. We answer these questions by combining morphology selection maps, interpretable driver attribution through pairwise effect landscapes, and composition–property landscapes conditioned on morphology that report physically meaningful low/intermediate/high regimes for key descriptors. The resulting framework provides actionable guidance for reducing harmful Fe‐IMCs in recycled Al–Si–Fe–Mn cast alloys and supports future data‐driven upcycling strategies.

## 2 Results

### 2.1 Interpreting Model Outputs as Microstructure Maps

As outlined at the end of the Introduction, our workflow is designed to answer three linked questions that metallurgists routinely ask when dealing with Fe‐IMCs: which morphology forms, why it forms, and how severe it is once it forms. Before reporting predictive metrics, we briefly clarify how the model outputs should be interpreted in physical terms, using familiar metallurgy analogies.

**FIGURE 4:**
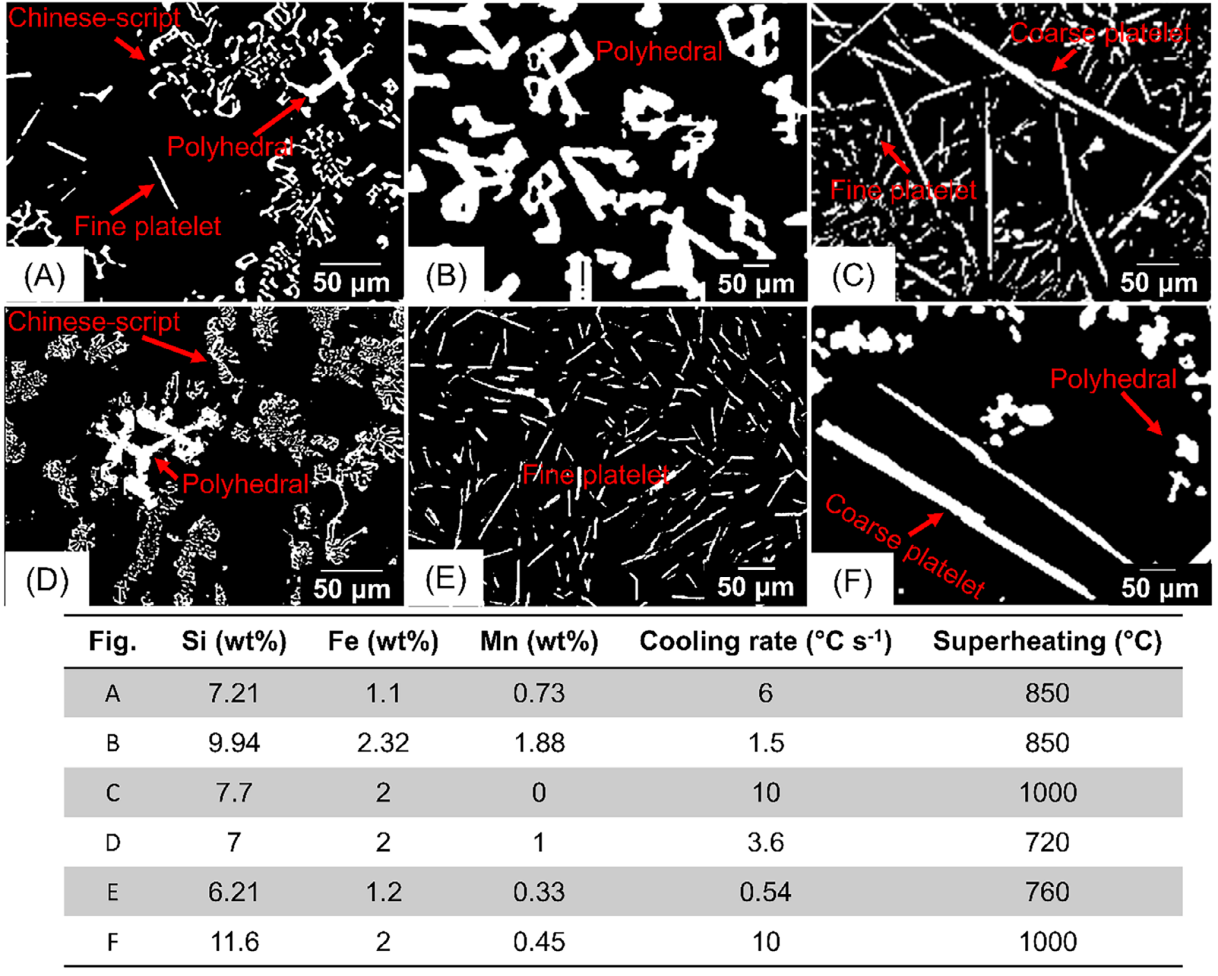
Representative Fe‐rich intermetallic morphologies used for model classification in recycled Al–Si–Fe–Mn cast alloys. Sample binary electron microscope micrographs and the corresponding alloy chemistry and processing conditions. The micrographs were sourced from the literature [17, 20, 21, 25, 26], please refer to the supporting information for the complete dataset. These micrographs illustrate the four Fe‐IMC morphology classes defined and predicted in this study: Chinese script, polyhedral, coarse platelet, and fine platelet. These classes constitute the target labels for the morphology probability models and serve as conditioning variables for the subsequent morphology‐dependent descriptor predictions. The distinction between coarse and fine platelet morphologies reflects differences in platelet thickness and length scale observed across the dataset.

The first‐stage models return a formation probability for each morphology class (Chinese script, polyhedral, coarse platelet, and fine platelet). In practice, a morphology probability map can be used to some extent analogous to a phase diagram, but it displays formation likelihoods rather than deterministic phase boundaries. This means that regions of high probability indicate conditions where a morphology is consistently observed in the training manifold, and gradients indicate transition zones where small changes in chemistry or processing shift the dominant morphology. Representative micrographs for the four morphology classes used throughout this work are shown in Figure 4, providing a visual anchor for non‐specialist readers.

The second‐stage models do not attempt to predict precise numerical values for descriptors such as area fraction, number density, size, and spacing. Instead, each descriptor is discretized into ordered regimes (low/intermediate/high) and predicted as an ordinal probability distribution. These ordinal outputs should be interpreted as regime maps, analogous to processing maps that delineate “low” vs “high” response regions, rather than as constitutive laws with point‐valued precision. Given the nature of sparse, noisy microstructural data, we intentionally aim at predicting well bounded regimes rather than point values. This approach not only emphasizes robust detection of transitions and dominant regimes and avoids overconfident regression, but also improves robustness against measurement variability arising from heterogeneous micrograph sources, by emphasizing consistent ordinal trends rather than exact numerical values.

Model performance is summarized with confusion matrices, which are simply contingency tables comparing predicted vs experimentally observed labels. Precision measures how many predicted positives are correct (“few false alarms”), whereas recall measures how many true positives are recovered (“few misses”). Importantly, our objective here is not perfect classification of every sample, but reliable identification of dominant morphology and descriptor regimes across composition–processing space.

(C) Conceptual illustration of the ordinal cumulative model used to predict morphology‐conditioned descriptor severity. Instead of predicting exact numerical values, the model learns a sequence of binary exceedance probabilities, first separating low from medium–high responses [*P*(*y* ≥ Medium)], and then separating low–medium from high responses [*P*(*y* ≥ High)]. The final class probabilities are reconstructed from these cumulative predictions, yielding an ordered regime‐level description of descriptor severity.

**FIGURE 5:**
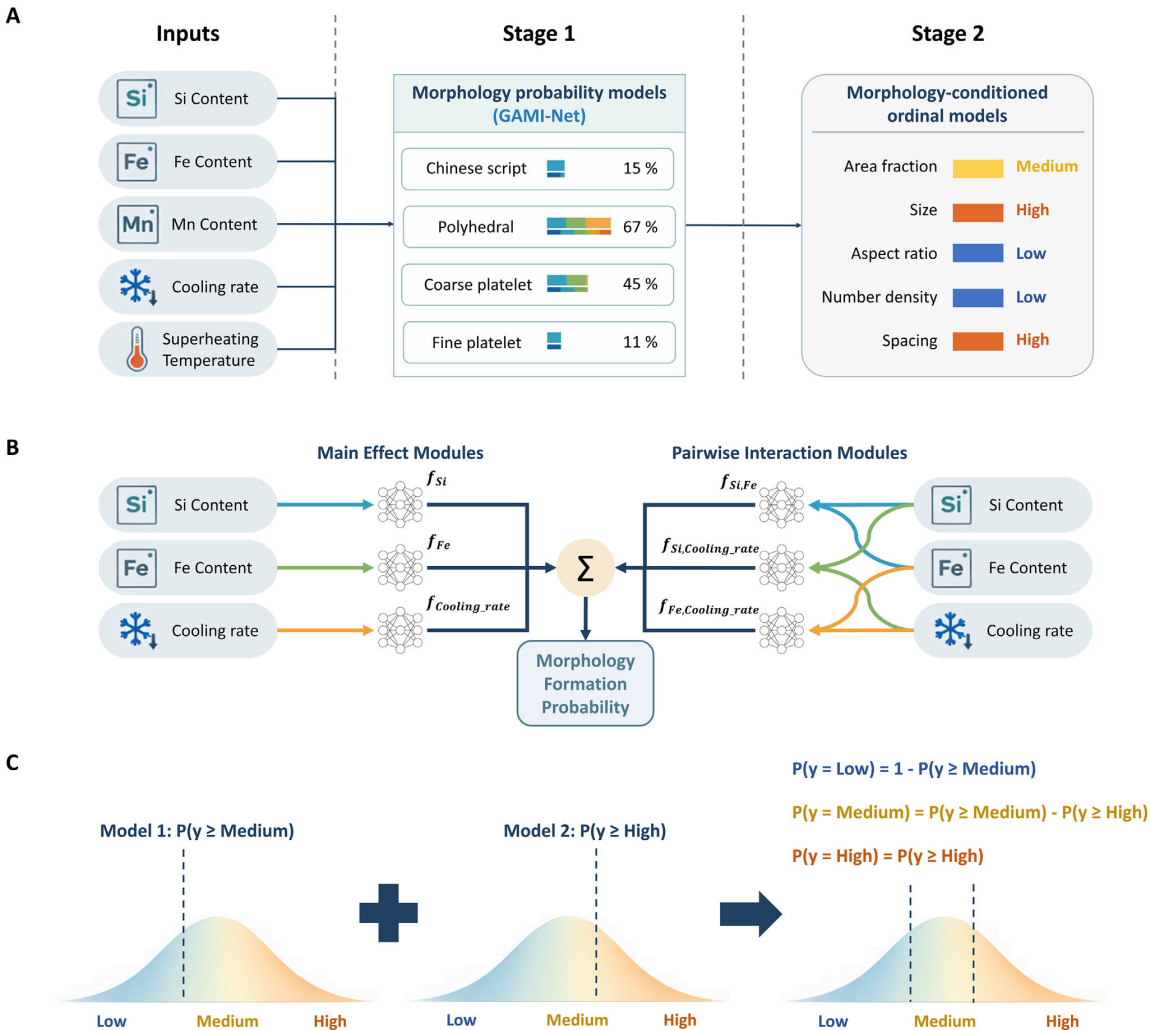
Conceptual overview of the two‐stage interpretable modeling framework used in this study. (A) Schematic illustration of the two‐stage prediction workflow linking alloy composition and processing to Fe‐intermetallic (Fe‐IMC) microstructure. (B) Interpretable structure of the morphology probability model based on a generalized additive framework with pairwise interactions (GAMI‐Net).

To help readers grasp the logic of the framework at a glance, Figure 5 provides a conceptual overview of the two‐stage modeling strategy, illustrating how alloy composition and processing conditions are first mapped to Fe‐intermetallic morphology probabilities using an interpretable additive model, and how morphology‐conditioned ordinal models subsequently translate these outcomes into physically meaningful low, intermediate, and high burden regimes for key microstructural descriptors.

### 2.2 Predictive Performance of Data‐Driven Microstructure Models

To assess how effectively the proposed framework captures processing–microstructure relationships for Fe‐IMCs in recycled Al–Si–Fe–Mn cast alloys, we evaluated (i) morphology‐type formation probability models and (ii) ordinal regime models for morphology descriptors (mean/max island aspect ratio, mean/max island area, mean/max island size, mean island circularity, mean branch thickness, area fraction, island number density, and median nearest‐neighbor distance (NND) of IMCs) [27, 28].

All models were trained on a dataset comprising 71 distinct combinations of alloy chemistry and thermal processing conditions, defined by variations in Si, Fe, and Mn contents, cooling rate, and superheating temperature. For descriptor prediction, the dataset was augmented by including the morphology type (Chinese script, polyhedral, coarse platelet, fine platelet) as an additional input, yielding 284 total samples. This augmentation reflects the physical fact that descriptor statistics are morphology‐conditioned, and it improves statistical efficiency without having to create artificial microstructure examples.

We trained four separate GAMI‐Net classifiers to predict the formation probability of each morphology type, where the acronym stands for ‘Generalized Additive Model with Interactions Network’. This architecture can capture individual feature contributions and pairwise interaction effects influencing the outcome, while maintaining the additive structure of classic Generalized Additive Models (GAMs). Here, it was specifically selected over conventional fully connected neural networks because it provides an inherently interpretable structure aligned with our goal of attributing morphology selection to specific compositional and processing couplings. While a standard neural network learns a single mixed, opaque mapping,

(1) $$\hat{y}=f\left(x_{1},x_{2},\text{…},x_{d}\right)$$

such that the influence of one variable (e.g., Mn) cannot be cleanly separated from others, GAMI‐Net represents the prediction as a sum of main effects and pairwise interactions,

(2) $$\hat{y}=\beta_{0}+\sum_{i=1}^{d}f_{i}\left(x_{i}\right)+\sum_{1\leq i<j\leq d}f_{i,j}\left(x_{i},x_{j}\right)$$

The terms *f_i_* (*x_i_*) quantify the isolated influence of each compositional or processing variable, whereas *f*_*i*,*j*_(*x_i_*,*x_j_*) capture the interaction between variable pairs. For readers less familiar with interpretable machine‐learning models, Equation (2) can be viewed as a data‐driven analogue of a response‐surface formulation, in which individual alloying elements or processing parameters contribute additively, and selected pairs capture physically meaningful couplings such as Fe–Mn chemistry or cooling‐rate–superheating interactions. Although ternary and higher‐order interactions are not represented explicitly, the resulting transparency enables a physically grounded interpretation of which couplings dominate morphology selection; these learned drivers are analyzed in a later section using pairwise effect landscapes.

**FIGURE 6:**
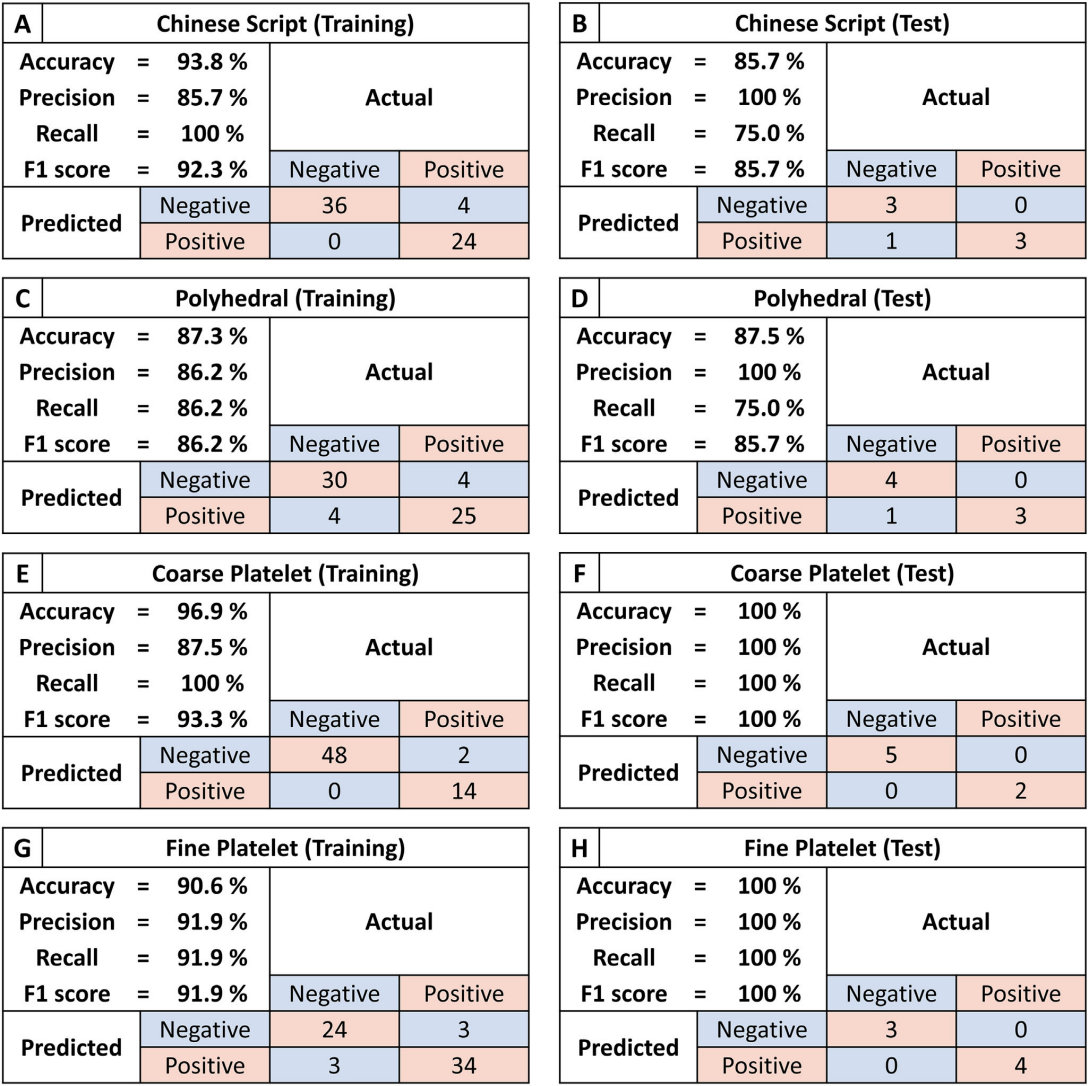
Confusion matrices and classification metrics for GAMI‐Net models predicting Fe‐intermetallic morphology formation. Confusion matrices summarizing the performance of GAMI‐Net classifiers for predicting the formation of four Fe‐intermetallic morphologies in recycled Al–Si–Fe–Mn cast alloys: (A,B) Chinese script, (C,D) polyhedral, (E,F) coarse platelet, and (G,H) fine platelet. Panels (A, C, E, G) show results for the training set, while (B, D, F, H) correspond to the independent test set. In each matrix, columns denote the experimentally observed morphology and rows the model prediction. Accuracy represents the fraction of correctly classified samples. Precision quantifies how many samples predicted as a given morphology are truly of that morphology (low false‐positive rate), whereas recall quantifies how many of the true cases are correctly identified (low false‐negative rate). The F1 score is the harmonic mean of precision and recall and balances these two aspects of the classification performance.

The predictive performance of the morphology classifiers is summarized by confusion matrices and standard metrics in Figure 6. Across all four morphologies, the models show consistently high fidelity, indicating that the dominant morphology regimes in composition–processing space are captured reliably despite the limited dataset size. For Chinese script and polyhedral morphologies, the test‐set performance yields perfect precision (no false positives) but only moderate recall. This means that the model is conservative in a positive sense: when it predicts that these types of morphology features are present it is almost always correct, although it sometimes misses true cases near transition regions. For coarse and fine platelet morphology cases, the test‐set metrics reach 100% in this dataset split. However, this should be interpreted with caution because the number of test samples for these minority classes has been rather limited. Overall, the modest train–test gaps support satisfactory generalizability within the explored training manifold, and the conservative behavior (high precision) is advantageous when the maps are used for microstructure‐risk screening.

To assess whether a neural‐network‐based model is necessary in the present case, given the moderate dataset size, we benchmarked the GAMI‐Net classifiers against simpler non‐neural baselines, including logistic regression with explicit pairwise interaction terms, random forest, and histogram‐based gradient boosting. The results are summarized in Table S1. Across all four morphology‐classification tasks, these baseline models achieve predictive performance broadly comparable to that of GAMI‐Net. For instance, identical test accuracies are observed for several morphologies, and logistic regression with interaction terms even yields a slightly higher F1 score for the Chinese‐script class. These findings indicate that competitive predictive accuracy can already be achieved with simpler models. Therefore, the motivation for employing GAMI‐Net in this work is not improved accuracy, but its ability to provide an explicit additive decomposition into main and pairwise effects, which is essential for the interpretability analyses presented in Sections 2.3 and 2.4.

To predict quantitative morphology descriptors under sparse, heterogeneous data, we employed ordinal cumulative models that treat each descriptor not as a continuous scalar but as an ordered category (low/intermediate/high). This choice is particularly well suited for microstructural variables with wide dynamic ranges and measurement uncertainty, where direct regression can overfit and produce misleading point predictions. In the ordinal cumulative formulation, the probability that a descriptor *y* exceeds a threshold *k* is modeled as

(3) $$P\;\left(y>k|x\right)=\sigma \left(\eta_{k}\left(x\right)\right)$$

where σ(·) is the logistic link and η_*k*_(*x*) is the predictor for the *k*‐th cumulative comparison. The probability of membership in an exact category is obtained by differencing adjacent cumulative probabilities,

(4) $$P\;\left(y=k|x\right)=P\left(y>k-1|x\right)-P\left(y>k|x\right)$$

This structure mirrors classical cumulative‐link models while preserving the intrinsic ordering of regimes, stabilizing learning when intermediate cases are sparse or near regime boundaries.

Descriptors such as area fraction and island number density are zero‐inflated, meaning more than half of the observations are exactly zero. In such cases, standard regression becomes unreliable because it is dominated by the large mass at zero and struggles to learn the physically relevant transitions among non‐zero responses. To address this, each target was discretized into three ordered bins: all zero‐valued samples were assigned to Bin 0, and non‐zero values were divided by percentile thresholds such that Bin 0 additionally contains the 0–33rd percentile, Bin 1 the 33–67th percentile, and Bin 2 the 67–100th percentile of the non‐zero subset. This binning preserves ordinal meaning (“zero/low” → “medium” → “high”) while mitigating imbalance.

A summary of ordinal‐model performance is provided in Table 2, and confusion matrices for all 11 descriptor models are shown in Figures S1–S6. Test accuracies span 71.4%–93.1%, indicating that the models capture the dominant descriptor trends as functions of composition, processing, and morphology type. Several descriptors show particularly strong predictability, including mean/max aspect ratio and spatial descriptors (area fraction, number density, and NND), consistent with these variables reflecting robust, regime‐level microstructural changes. Size‐ and area‐related descriptors achieve moderate‐to‐high test accuracy, and the highest‐response regime (Bin 2) is consistently recovered with high F1 scores, indicating strong sensitivity to conditions that produce especially large or abundant intermetallic features. The most challenging target is mean island circularity, which shows lower performance in the low‐response regime, plausibly because circularity is affected by multiple coupled mechanisms (growth, fragmentation, and coalescence) that are not fully parameterized by the five processing/chemistry inputs in the present dataset.

**TABLE 2:** Summary of predictive performance of the ordinal cumulative models for 11 morphology descriptors of Fe‐intermetallic phases (Fe‐IMCs). The table reports training and test accuracies, along with bin‐wise F1 scores for the three ordered output categories (Bin 0: Zero or low response; Bin 1: Medium response; Bin 2: High response).

Target	Train Accuracy (%)	Test Accuracy (%)	Test F1(Bin 0) (%)	Test F1(Bin 1) (%)	Test F1(Bin 2) (%)
Mean island aspect ratio	91.5	91.7	100	80.0	88.9
Max island aspect ratio	81.1	91.7	85.7	88.9	100
Mean island area (µm^2^)	84.0	83.3	80.0	66.7	100
Max island area (µm^2^)	75.5	75.0	80.0	57.1	85.7
Mean island size (µm)	79.3	75.0	75.0	75.0	75.0
Max island size (µm)	78.3	83.3	80.0	80.0	88.9
Mean island circularity	75.9	71.4	50.0	66.7	100
Mean branch thickness (µm)	90.7	85.7	100	80.0	80.0
Area fraction (%)	90.6	89.7	93.6	66.7	75.0
Island number density (/mm^2^)	96.1	93.1	97.7	50.0	90.9
Median nearest‐neighbor distance (NND) of IMCs (µm)	92.1	87.5	96.3	81.8	92.0

Taken together, the GAMI‐Net morphology classifiers and the ordinal cumulative descriptor models provide a reliable and interpretable mapping from alloy chemistry and thermal processing conditions to both morphology selection and morphology‐conditioned burden regimes. This validated foundation supports the subsequent sections, where the trained models are used to generate (i) model‐derived morphology probability maps, (ii) driver attribution through pairwise effect landscapes, and (iii) ordinal composition–property landscapes that quantify how microstructural burden is redistributed within each morphology.

### 2.3 Model‐Derived Probability Maps for Fe‐IMC Morphology Selection

Beyond aggregate performance metrics, we examined whether the trained GAMI‐Net classifiers recover physically meaningful morphology selection landscapes across the composition and processing space of recycled Al–Si–Fe–Mn cast alloys. The resulting model‐derived probability maps can be interpreted in the same spirit as composition–processing maps commonly used in alloy design. Rather than delineating equilibrium phase boundaries, these maps indicate the likelihood of observing a given Fe‐rich intermetallic morphology under specific combinations of alloy chemistry and solidification conditions within the experimentally sampled domain. High‐probability regions therefore identify dominant morphology fields, whereas smooth gradients and diagonal bands mark transition corridors in which small changes in composition or processing shift the preferred morphology.

To construct these maps, five input variables (Si, Fe, and Mn contents, cooling rate, and superheating temperature) were considered. For each visualization, three variables were fixed at representative anchor values, while the remaining two were varied systematically within their respective training ranges. The fixed values were selected to represent statistically representative conditions within the dataset. In particular, a cooling rate of 10°C s^−1^ corresponds to the most frequent value, while a superheating temperature of 800°C lies between the two dominant superheat clusters (720°C and 850°C) and is close to the dataset median (760°C), thereby providing a balanced reference condition.

**FIGURE 7:**
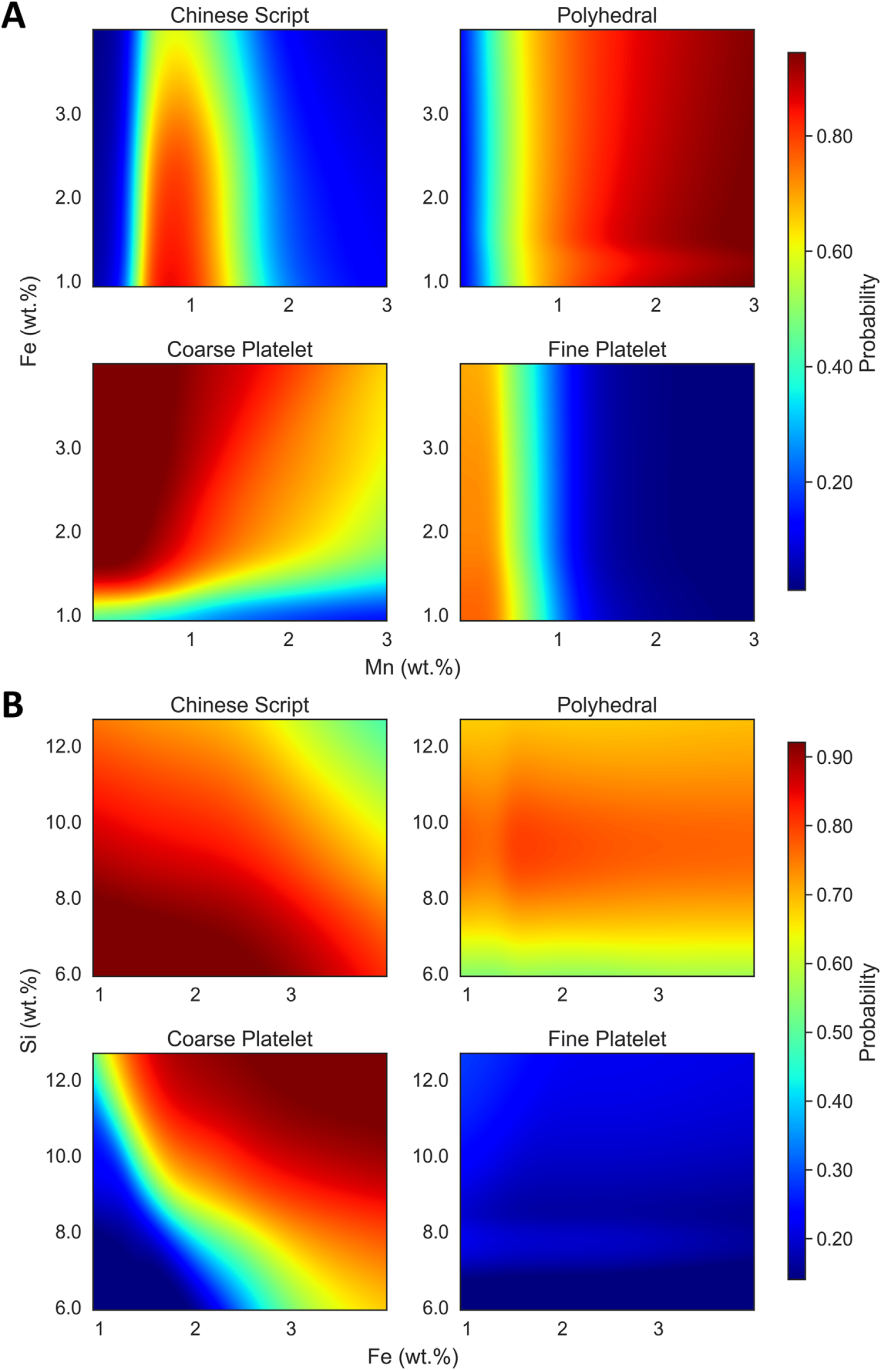
Model‐derived probability maps for the formation of Fe‐rich intermetallic compound (Fe‐IMC) morphologies as a function of alloy composition. (A) Fe and Mn contents at fixed Si = 11 wt.%, cooling rate = 10°C s^−1^, and superheating temperature = 800°C. (B) Fe and Si contents at fixed Mn = 1 wt.%, cooling rate = 10°C s^−1^, and superheating temperature = 800°C. Separate panels show the predicted formation probability of Chinese script, polyhedral, coarse platelet, and fine platelet morphologies obtained from the trained GAMI‐Net models. Color represents probability on a scale from 0 to 1, with warmer colors indicating higher likelihood.

**FIGURE 8:**
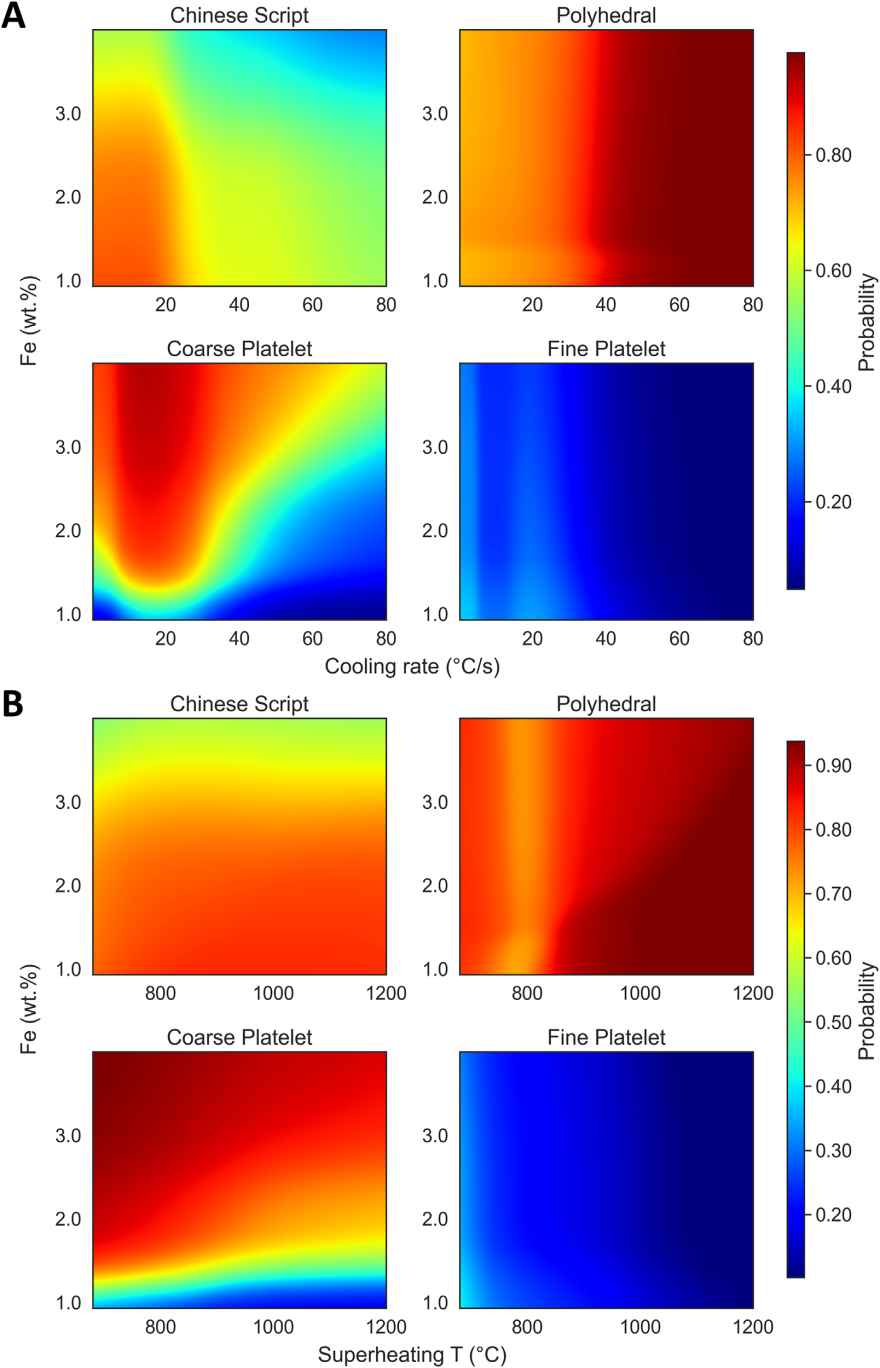
Model‐derived probability maps illustrating the influence of processing parameters on Fe‐rich intermetallic compound (Fe‐IMC) morphology selection. (A) Fe content and cooling rate at fixed Si = 11 wt.%, Mn = 1 wt.%, and superheating temperature = 800°C. (B) Fe content and superheating temperature at fixed Si = 11 wt.%, Mn = 1 wt.%, and cooling rate = 10°C s^−1^. For each condition, the predicted probabilities of Chinese script, polyhedral, coarse platelet, and fine platelet morphologies are shown in separate panels. Color indicates the predicted probability (0–1), with warmer colors corresponding to a higher likelihood of the given morphology.

This procedure is analogous to reading a phase diagram on a selected section through a higher‐dimensional space. For each two‐dimensional slice, a 100 × 100 grid was sampled and the predicted formation probability of each Fe‐intermetallic morphology (Chinese script, polyhedral, coarse platelet, and fine platelet) was evaluated. The resulting probability fields are presented as heatmaps in Figures 7 and 8. These maps summarize in‐domain interpolations learned from the available dataset and should therefore be interpreted as learned trends rather than extrapolations beyond the training ranges of Fe (0.93–4.00 wt.%), Mn (0–3 wt.%), Si (5.93–12.70 wt.%), cooling rate (0.1–80°C s^−1^), and superheating temperature (680°C–1200°C).

The Fe–Mn probability map constructed at fixed Si = 11 wt.%, cooling rate = 10°C s^−1^, and superheating temperature = 800°C (Figure 7A) identifies Mn as the primary discriminator between compact and platelet‐like morphologies, while Fe governs the overall tendency toward platelet formation, consistent with prior experimental observations that Mn additions promote transformation from platelet‐like Fe‐IMCs toward more compact α‐type morphologies, whereas increasing Fe stabilizes platelet‐forming regimes [29]. Chinese script morphology forms a pronounced probability band at intermediate Mn contents of approximately 0.6–1.2 wt.%, with likelihood decreasing at both lower and higher Mn levels. This nonmonotonic dependence reveals a relatively narrow composition window in which coupled Fe–Mn chemistry favors script‐like growth. Within this window, increasing Fe gradually suppresses the Chinese script field, consistent with a shift toward Fe‐rich platelet regimes at higher Fe contents.

Polyhedral morphology, by contrast, increases monotonically with the Mn content across the full Fe range and establishes a broad high‐probability field at Mn contents above roughly 1.5–2.0 wt.%, with comparatively weak sensitivity to Fe, reproducing the well‐known Mn‐driven stabilization of compact α‐type Fe‐containing intermetallic morphologies in recycled Al–Si alloys [30]. The model therefore partitions the Mn‐controlled compact‐morphology regime into two distinct subdomains: intermediate Mn favors Chinese script, whereas higher Mn content stabilizes polyhedral morphologies. This separation has direct practical impact: it indicates that Mn additions do not act as a single‐direction control parameter but instead drive a sequence of morphology transitions from platelet‐like structures to Chinese script and, at sufficiently high Mn content, further toward polyhedral shapes.

The platelet‐like morphologies occupy complementary regions of the Fe–Mn space. Coarse platelet probability increases strongly with Fe and decreases with Mn, producing a diagonal transition corridor from high Fe–low Mn to low Fe–high Mn. This structure reflects an antagonistic interaction between Fe and Mn, whereby Mn progressively counteracts the platelet‐promoting influence of Fe and shifts the morphology boundary toward higher Fe contents. Fine platelet morphology exhibits an even stronger sensitivity to Mn, with predicted probabilities collapsing rapidly once Mn exceeds approximately 0.5–1.0 wt.%. Under the fixed Si and processing conditions of Figure 7A, fine platelets are therefore primarily associated with low‐Mn compositions. Taken together, the Fe–Mn map provides a concise morphology selection landscape in which Mn suppresses platelet formation while Fe promotes it unless offset by sufficient Mn additions.

The Fe–Si probability map generated at fixed Mn = 1 wt.%, cooling rate = 10°C s^−1^, and superheating temperature = 800°C (Figure 7B) further clarifies the role of Si in modulating morphology selection. Chinese script morphology is favored at relatively low Fe and Si contents, with probabilities highest at Fe below approximately 3 wt.% and Si below approximately 9 wt.%. Inversely, Chinese script morphology decreases as either of these variables increases. Polyhedral morphology shows a clearer dependence on Si, maintaining moderate to high probabilities at Si contents above approximately 8 wt.% with limited sensitivity to Fe, in line with previous experimental reports that Si content can shift the relative stability and morphology of Fe‐rich intermetallic compounds under fixed Mn‐bearing conditions [31]. In contrast, coarse platelet probability increases with both Fe and Si, forming a high‐probability field at Fe above approximately 2 wt.% and Si above approximately 9 wt.%, also consistent with prior studies showing that elevated Fe together with sufficient Si promotes the formation and persistence of platelet‐like Fe‐rich intermetallic compounds in cast Al–Si alloys [32]. At fixed Mn content and moderate cooling, Si therefore acts as a reinforcing factor that stabilizes the coarse platelet regime when the Fe content is elevated.

Fine platelet morphology remains uniformly unlikely across the Fe–Si plane under these conditions. This does not imply that fine platelets cannot form, but rather that their dominant probability field lies elsewhere in the multidimensional composition–processing space, such as at lower Mn contents or under different thermal conditions. This observation is consistent with the strong Mn sensitivity revealed in the Fe–Mn maps and highlights the importance of multidimensional probability mapping for disentangling competing morphology selection mechanisms.

Figure 8 isolates the influence of processing variables by mapping morphology probabilities in the Fe–cooling‐rate and Fe–superheating‐temperature planes while holding Si and Mn fixed at 11 wt.% and 1 wt.%, respectively. In the Fe–cooling‐rate map (Figure 8A), Chinese script morphology occupies a high‐probability field at relatively low cooling rates, typically below approximately 25°C s^−1^, and decreases progressively as cooling rate increases, consistent with the general metallurgical expectation that slower solidification provides more time for the development and competitive coarsening of compact Fe‐IMC morphologies [33]. This decline is accentuated at higher Fe contents, indicating that under these compositional conditions the model associates Chinese script formation with slower solidification kinetics that allow sufficient time for morphological development and coarsening.

Polyhedral morphology remains prevalent across most of the Fe–cooling‐rate space and displays only weak sensitivity to cooling rate, suggesting that at Mn = 1 wt.% it is comparatively robust against kinetic variations within the sampled domain. Coarse platelet morphology, however, exhibits a structured dependence on cooling rate, with probabilities peaking at intermediate cooling rates of approximately 10–30°C s^−1^ when Fe exceeds roughly 2 wt.%. Lower probabilities are observed at both slower and faster cooling extremes, forming a ridge‐like probability field. This behavior indicates that platelet formation arises from a balance between compositional driving forces and kinetic constraints: slow cooling favors compact morphologies, whereas very rapid cooling suppresses platelet growth pathways. Fine platelet morphology remains unlikely throughout this plane, with only a modest increase at low cooling rates, reinforcing the conclusion that its formation is primarily chemistry‐controlled rather than kinetically driven under the present composition anchors.

The Fe–superheating‐temperature map (Figure 8B) reveals a distinct processing sensitivity. Chinese script probability varies only weakly with superheating temperature, with Fe again acting as the dominant suppressor at higher concentrations. Polyhedral morphology, in contrast, shows a pronounced dependence on superheating, achieving its highest probabilities at elevated superheating temperatures above approximately 900°C across a broad Fe range and exhibiting a local minimum near 800°C, which is qualitatively consistent with prior reports that melt thermal history and superheating influence Fe‐IMC nucleation pathways and the resulting morphology selection [34]. This trend suggests that melt thermal history influences morphology selection even at fixed composition, likely by modifying effective nucleation conditions or the dissolution state of pre‐existing intermetallic phases as reflected in the training data.

Coarse platelet probability increases with Fe but decreases systematically with increasing superheating temperature, producing a diagonal transition corridor, in agreement with earlier studies showing that sufficiently high superheating can suppress coarse platelet‐promoting pathways and shift the microstructure toward more compact Fe‐containing morphologies [35]. Higher superheating thus partially counteracts the platelet‐promoting effect of elevated Fe. Fine platelet probability remains low throughout this plane and decreases further at higher superheating temperatures, consistent with the broader observation that, for the selected composition anchors, fine platelets do not constitute a dominant morphology field within the model‐informed domain.

Taken together, Figures 7 and 8 demonstrate that the trained GAMI‐Net models provide more than discrete classification outcomes. They yield continuous probability landscapes that delineate dominant morphology fields and transition corridors across the multidimensional composition–processing space. Several physically interpretable trends emerge. Mn acts as a powerful suppressor of platelet morphologies and promotes compact forms, with a transition from Chinese script at intermediate Mn to polyhedral at higher Mn. Elevated Fe drives the system toward platelet formation, but this tendency can be mitigated through increased Mn content, higher superheating temperatures, or cooling rates that deviate from the intermediate regime most favorable to coarse platelet growth. Silicon further amplifies the coarse platelet regime when Fe is high at Mn = 1 wt.%, whereas polyhedral morphology benefits more generally from increased Si content.

The presence of diagonal boundaries and banded probability regions, rather than simple threshold behavior along single variables, highlights that Fe‐intermetallic morphology selection arises from coupled compositional and kinetic interactions. Capturing these structured effects in an interpretable form is a central advantage of the GAMI‐Net framework and establishes the foundation for the subsequent analysis of learned main and pairwise interaction effects.

### 2.4 Pairwise Effect Landscapes Reveal the Driving Feature Couplings Behind Morphology Selection

The probability maps in Section 2.3 visualize how the predicted likelihood of each Fe‐IMC morphology varies on selected two‐dimensional sections through the five‐dimensional composition–processing space, where three variables are held at representative anchor values. Such maps are useful for delineating morphology fields and transition corridors, but they do not by themselves identify which feature couplings are responsible for the model's decisions. In particular, trends observed on fixed‐condition slices can conflate multiple contributions (individual feature effects and multiple interactions) and may shift depending on the chosen anchors, analogous to how a single isothermal or isopleth section can obscure the controlling thermodynamic interactions in a higher‐dimensional phase space.

To extract the underlying drivers in a condition‐independent manner, we leveraged the structured additive decomposition of GAMI‐Net to construct pairwise total effect landscapes. This analysis addresses a common limitation of traditional parametric studies, where trends observed at fixed conditions may obscure the true controlling interactions. By marginalizing over all other variables (i.e., averaging over the distributions of the remaining inputs), the pairwise effect landscapes expose which couplings consistently govern morphology selection across the broader design space sampled by the experiments.

For a given feature pair (*x_i_*,*x_j_*), the pairwise total effect is defined as

(5) $$z_{i,j}\;\left(x_{i},x_{j}\right)=f_{i}\;\left(x_{i}\right)+f_{j}\left(x_{j}\right)+f_{i,j}\left(x_{i},x_{j}\right)$$

where *f_i_* and *f_j_* are the learned main effects and *f*_*i*,*j*_ is their learned interaction. In physical terms, *z*_*i*,*j*_ can be viewed as a response‐surface section for a selected variable pair that retains both the isolated contributions of each variable and their coupling, while averaging out (“marginalizing”) the influence of the remaining inputs. Positive values of *z*_*i*,*j*_ indicate that the selected pair promotes the formation probability of the corresponding morphology, whereas negative values indicate suppression. Each landscape was evaluated on a regular 100 × 100 grid spanning the training range of the corresponding feature pair.

To enable direct comparison across morphologies and across feature pairs, the pairwise effects were normalized by the standard deviation of *z* computed across all possible feature pairs within each morphology model. A shared color scale, defined from the pooled fifth–95th percentile of all normalized values, was applied across all panels, such that every map spans the same visual range. This consistent scaling allows the relative strength of different pairwise drivers to be compared directly rather than interpreted in isolation.

**FIGURE 9:**
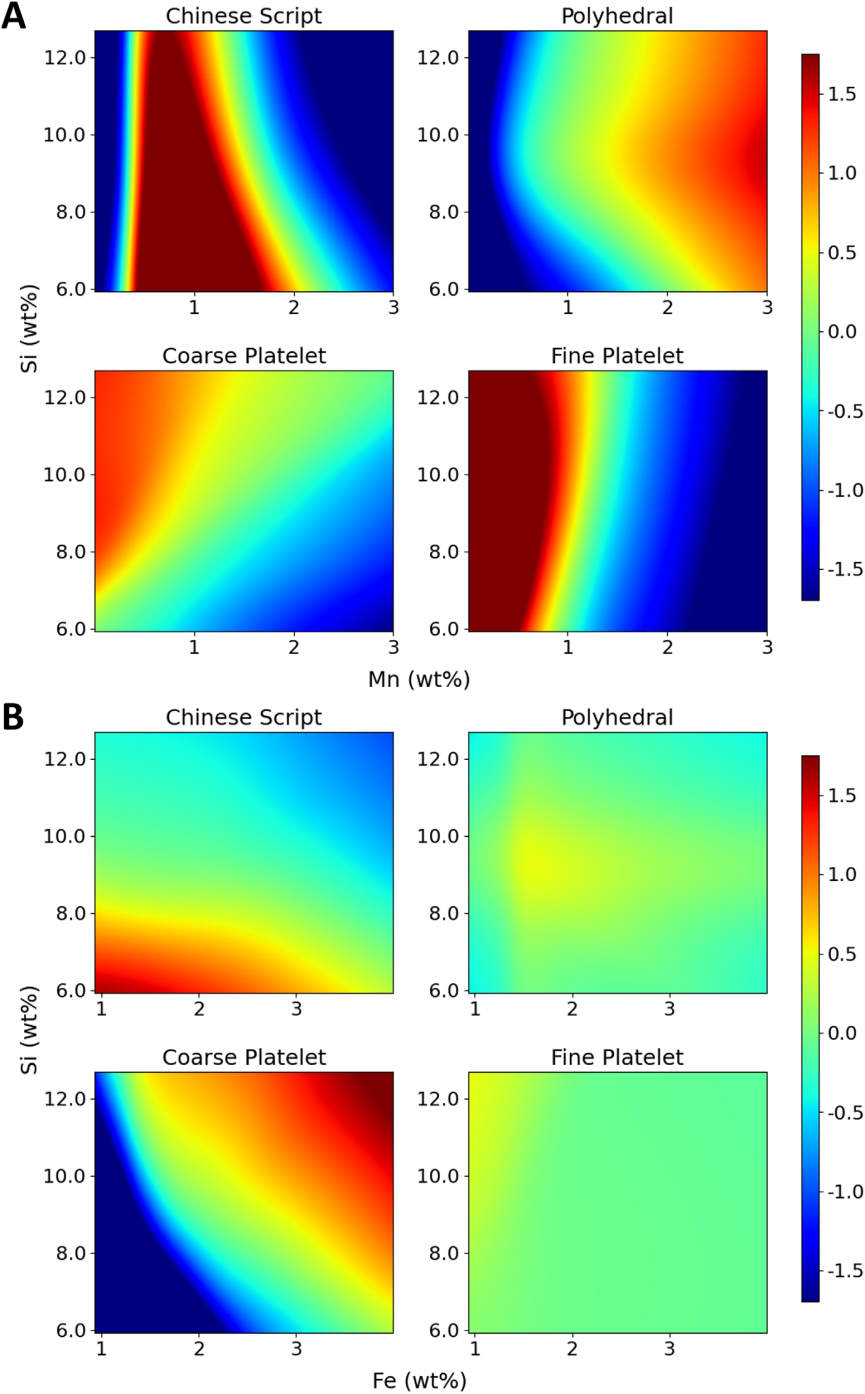
Pairwise total effect landscapes *z*_*i*,*j*_ = *f_i_*  + *f_j_* + *f*_*i*,*j*_ illustrating the isolated contribution of compositional feature couplings to Fe‐rich intermetallic compound (Fe‐IMC) morphology selection. (A) Fe–Mn and (B) Fe–Si total effects are shown for Chinese script, polyhedral, coarse platelet, and fine platelet morphologies. The total effect was evaluated on a 100 × 100 grid spanning the training range of each feature pair, while marginalizing over all remaining inputs. Positive values indicate that the corresponding feature pair supports formation of the given morphology, whereas negative values indicate suppression. To enable comparison across morphologies and feature pairs, all values were normalized by the standard deviation of *z* across all possible feature pairs within each model, and a common color scale (fifth–95th percentile, z∈[−1.6,1.6]) was applied to all panels.

Figure 9 summarizes the dominant compositional couplings revealed by the pairwise landscapes. For Chinese script morphology, the Fe–Mn landscape exhibits a pronounced positive window centered at intermediate Mn contents, with strong suppression at both Mn‐deficient and Mn‐rich extremes. Within this Mn band, increasing Fe reduces the pairwise support. This pattern closely echoes the band‐shaped probability maximum in Figure 7A and confirms that it originates from a primary Fe–Mn coupling, rather than being an artifact of fixed‐condition slicing. This interpretation is consistent with prior metallurgical studies identifying the Fe–Mn composition balance as a principal control parameter for guiding the Fe‐IMC morphology transition in Al–Si–Fe–Mn alloys [36]. In the Fe–Si composition space, the Chinese‐script pairwise contribution decreases monotonically with increasing Fe and Si contents, indicating that concurrent enrichment in these elements acts as a coherent suppressor of script‐like formation. This provides an interpretable basis for the contraction of the Chinese‐script probability field at high Fe and high Si in Figure 7B.

Polyhedral morphology displays a different compositional signature. In Fe–Mn space, Mn acts as an effective switch: low Mn yields strongly negative contributions, whereas increasing Mn progressively supports polyhedral formation across the Fe range. Fe plays a comparatively secondary role, introducing only a modest preference at intermediate Fe. In contrast, the Fe–Si pairwise landscape for polyhedral remains near zero across most of the domain, indicating that Fe–Si coupling is not a primary determinant of polyhedral selection within the learned model. This interpretability check is important when reading Figure 7B: any apparent Si‐related variations in polyhedral probability are more plausibly mediated through other variables or more influential feature pairs, rather than through a direct Fe–Si interaction.

Coarse platelet morphology exhibits the clearest antagonistic compositional coupling, consistent with the established role of Mn in counteracting Fe‐driven stabilization of platelet‐like Fe‐rich intermetallics [37]. Its Fe–Mn landscape increases with Fe and decreases with Mn, forming a diagonal transition that highlights Mn as a systematic counterbalance to the platelet‐promoting influence of Fe. In Fe–Si space, the pairwise landscape shows a pronounced synergistic structure: concurrent increases in Fe and Si strongly support coarse platelet formation. Together, these two couplings explain why coarse platelets concentrate in high‐Fe/low‐Mn regions and why their probability field expands toward higher Si in Figure 7B.

Fine platelet morphology is dominated by Mn dependence. The Fe–Mn pairwise support decreases nearly monotonically as Mn increases, with only weak sensitivity to Fe. Its Fe–Si pairwise landscape remains largely flat near zero values, indicating that Si does not act as a major pairwise driver of fine‐platelet selection. This aligns with the uniformly low fine‐platelet probabilities across Fe–Si space in Figure 7B and reinforces the conclusion that fine‐platelet formation is primarily associated with low‐Mn chemistries.

**FIGURE 10:**
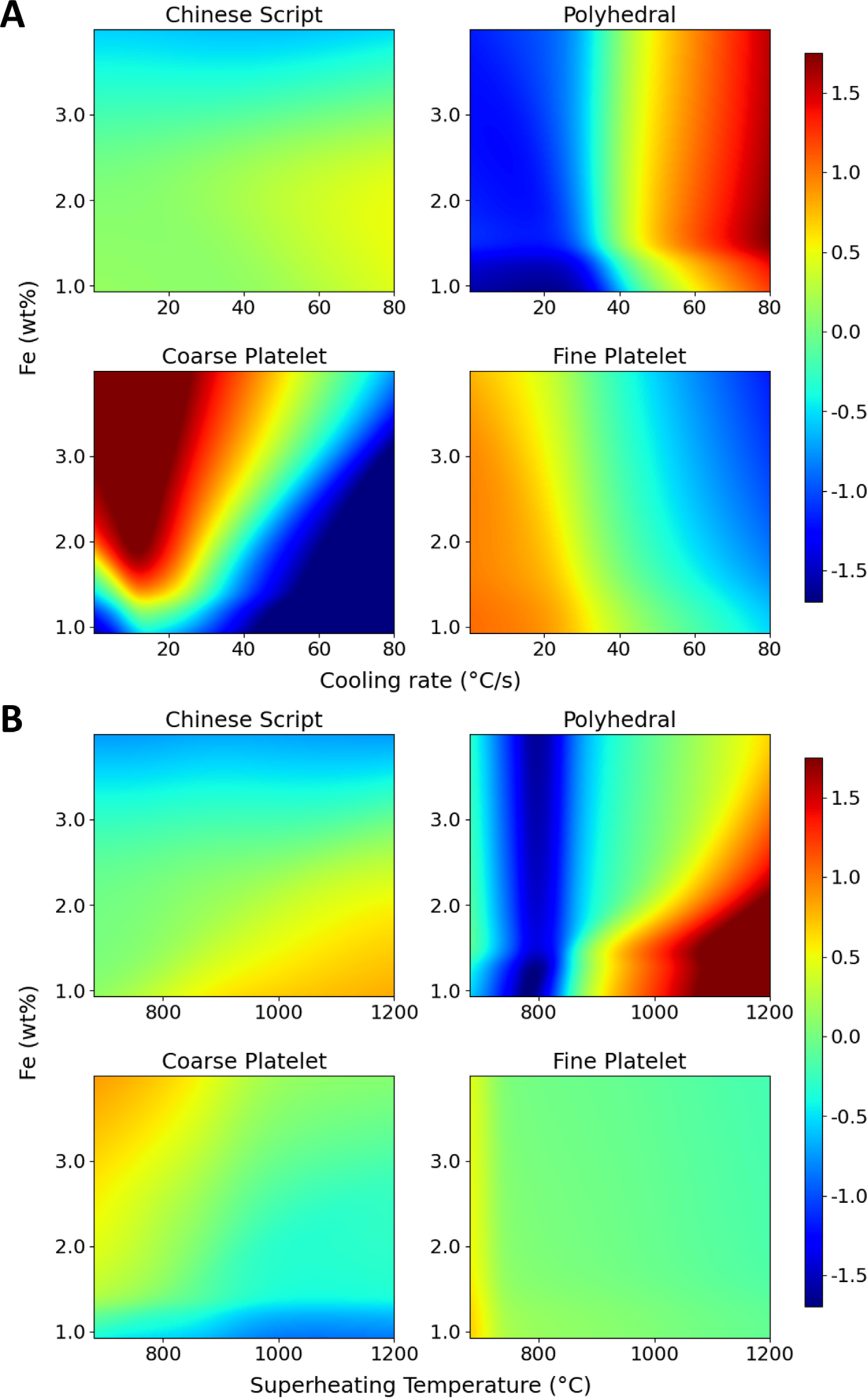
Pairwise total effect landscapes *z*_*i*,*j*_ = *f_i_*  + *f_j_* + *f*_*i*,*j*_ highlighting the role of processing–composition couplings in Fe‐IMC morphology selection. (A) Fe–cooling‐rate and (B) Fe–superheating‐temperature total effects are shown for Chinese script, polyhedral, coarse platelet, and fine platelet morphologies. The total effect represents the combined contribution of the individual feature effects and their interaction, evaluated on a regular 100 × 100 grid over the training range of each pair while marginalizing over all other inputs. Values were normalized by the standard deviation of *z* across all feature pairs, and a shared color scale (z∈[−1.6,1.6], fifth–95th percentile) was used for all panels.

Figure 10 highlights how processing variables can dominate morphology selection for specific classes and do so in ways that are not captured by simple monotonic trends. For Chinese script, the Fe–cooling‐rate pairwise effect is comparatively weak and varies smoothly, indicating that cooling rate plays a secondary role relative to compositional couplings. This is consistent with Figure 8A, where Chinese‐script probability decreases gradually with cooling rate without sharp transition corridors.

Polyhedral morphology shows some of the strongest processing‐driven couplings in the entire model set, which agrees with prior reports that compact α‐type Fe‐IMCs are highly sensitive to thermal history and solidification conditions [34]. The Fe–cooling‐rate landscape increases strongly with cooling rate, indicating robust support for polyhedral formation at high cooling rates across a broad Fe range. Even more distinctive is the Fe–superheating landscape, which exhibits a threshold‐like response: polyhedral support is reduced at intermediate superheating temperatures and then rises sharply once superheating exceeds approximately 900°C–1000°C, approaching the highest positive contributions at the upper end of the training domain. This pronounced nonlinearity provides an interpretable mechanism for the superheating sensitivity observed in Figure 8B and identifies melt thermal history as a primary lever for polyhedral selection within the learned model.

Coarse platelet morphology exhibits an opposing processing signature. Its Fe–cooling‐rate landscape peaks at intermediate cooling rates rather than increasing monotonically, indicating that platelet‐favorable conditions reflect a balance of kinetic effects rather than a single directional rate dependence. This ridge‐like structure explains the probability maximum at moderate cooling rates in Figure 8A and suggests that both slower and faster cooling shift the system away from the pathway that most strongly supports coarse platelet development. In the Fe–superheating landscape, pairwise support decreases with increasing superheating and increases with Fe, forming a diagonal boundary. This boundary indicates that higher superheating can partially offset the platelet‐promoting effect of elevated Fe, which is consistent with the diagonal transition corridor shown in Figure 8B.

Fine platelet morphology shows strong suppression with increasing cooling rate but only weak sensitivity to superheating, reinforcing the conclusion that fine‐platelet selection is governed primarily by chemistry (especially Mn) and cooling kinetics, with limited influence of melt superheating within the explored domain.

Taken together, the pairwise effect landscapes provide a compact and interpretable ranking of the dominant couplings that govern morphology selection, clarifying why different morphologies respond to different levers. Chinese script selection is governed primarily by compositional windows and suppressors in Fe–Mn and Fe–Si space. Coarse platelet selection is driven by antagonistic Fe–Mn coupling, synergistic Fe–Si coupling, and a non‐monotonic dependence on cooling rate, with superheating acting as a stabilizing suppressor at fixed Fe. Polyhedral morphology is distinguished by strong processing control, particularly through cooling rate and a threshold‐like dependence on superheating, while exhibiting comparatively weak Fe–Si coupling. Fine platelet selection is largely controlled by Mn content and cooling rate, with minimal pairwise sensitivity to Si and superheating.

This interpretability layer bridges the probability maps in Section 2.3 and the morphology‐conditioned composition–property landscapes presented next in Section 2.5 by explicitly identifying which couplings shift morphology fields and therefore determine where specific microstructural burden signatures can emerge.

### 2.5 Ordinal Model Derived Composition–Property Landscapes of Fe‐IMCs

The probability maps (Section 2.3) and pairwise effect landscapes (Section 2.4) establish which Fe‐IMC morphology is favored and which couplings drive the transitions. The next question concerns severity, that is, how much of the Fe‐IMC phase forms and what morphological features it exhibits. We introduce the term “intermetallic burden” to denote how a given alloy variant accommodates Fe‐IMCs in its microstructure, specifically in terms of extent, population, shape, size, and spacing. To address this, we constructed morphology‐conditioned composition–property landscapes using the trained ordinal cumulative models. Conceptually, this approach resembles classifying microstructures into acceptable, marginal, and critical regimes, rather than attempting to predict an exact numerical descriptor that may not be experimentally robust under sparse data.

For each descriptor, three of the five continuous inputs were fixed at representative anchor values while the remaining two were varied across their training ranges on a 100 × 100 grid. For a selected morphology type (Chinese script, polyhedral, coarse platelet, or fine platelet), the ordinal model returns a probability vector over three ordered response bins. Each grid point is summarized by the expected bin index,

(6) $$E\;\left[\mathrm{bin}\right]=\sum_{k=0}^{2}k\cdot P\left(\mathrm{bin}=k\right)\;,$$

which yields a continuous surface that preserves the ordinal meaning of low, intermediate, and high response regimes without implying the precision of direct regression. In the maps shown below, this expected ordinal response is termed E[·] for brevity, and gradients are interpreted primarily as transitions between regimes rather than as absolute quantitative predictions.

Because several descriptors are undefined or uninformative when a certain morphology does not form, we masked low‐likelihood regions prior to interpretation. Specifically, the corresponding GAMI‐Net morphology probability model was used to identify grid points with predicted formation probability below 0.5, which were greyed out in the descriptor maps. This step prevents over‐interpretation outside the supported data manifold and keeps descriptor landscapes consistent with the morphology selection domains identified in Section 2.3. Accordingly, the discussion focuses on trends within the formed regions, while masked areas are treated as non‐actionable regions for descriptor interpretation.

**FIGURE 11:**
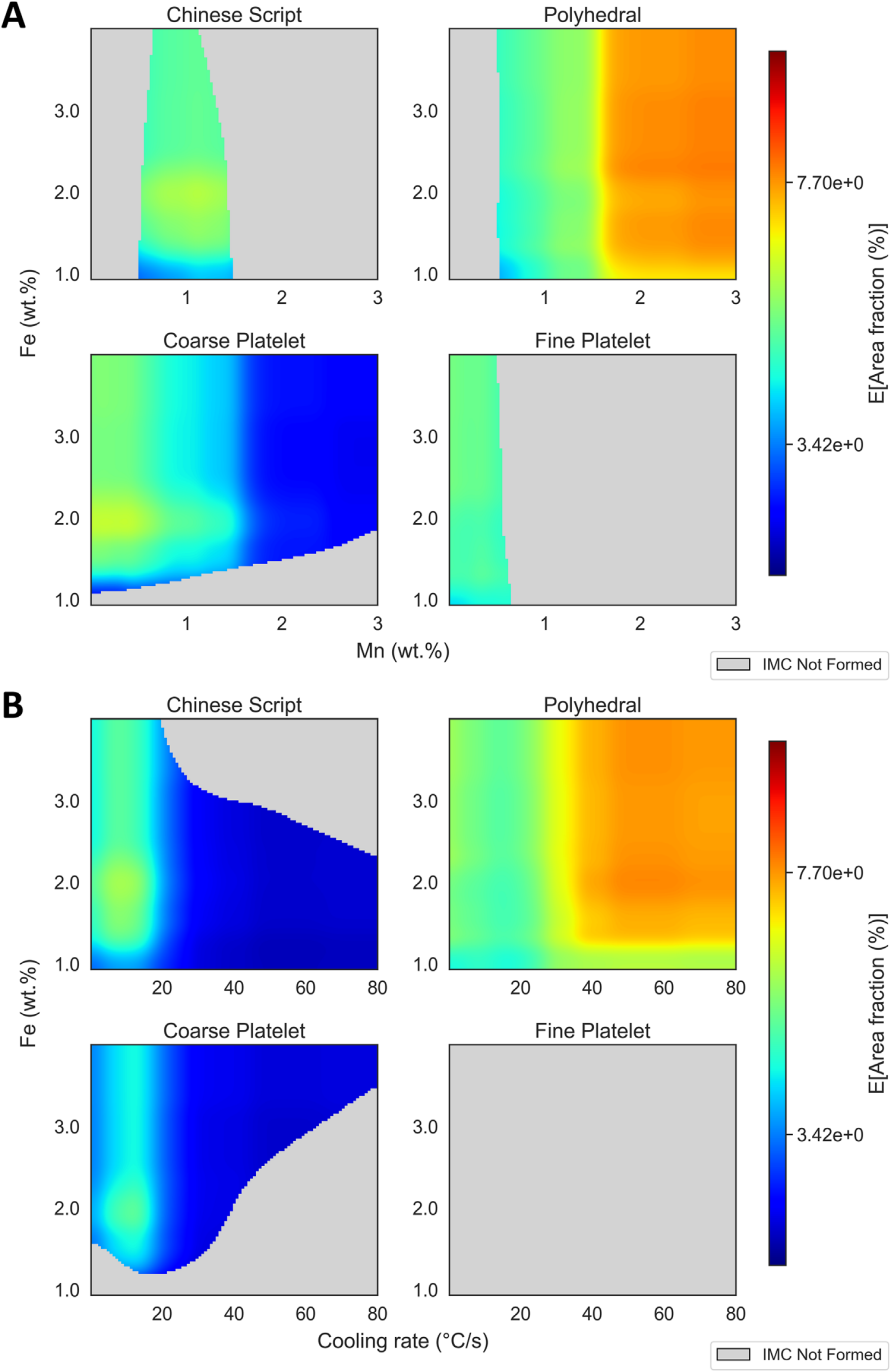
Composition–property maps showing the expected area fraction of Fe‐rich intermetallic compounds (Fe‐IMCs), E[Areafraction(%)], predicted by the ordinal cumulative models. (A) Fe and Mn contents at fixed Si = 11 wt.%, cooling rate = 10°C s^−1^, and superheating temperature = 800°C. (B) Fe content and cooling rate at fixed Si = 11 wt.%, Mn = 1 wt.%, and superheating temperature = 800°C. Separate panels correspond to Chinese script, polyhedral, coarse platelet, and fine platelet morphologies. Color represents the expected ordinal response level, obtained as the probability‐weighted expectation of the predicted bins, while grey regions indicate conditions where the corresponding morphology is not expected to form based on the GAMI‐Net formation probability (probability < 0.5).

Figure 11 summarizes expected area fraction regimes on two representative slices: Fe–Mn at fixed Si = 11 wt.%, cooling rate = 10°C s^−1^, and superheating temperature = 800°C (Figure 11A), and Fe–cooling‐rate at fixed Si = 11 wt.%, Mn = 1 wt.%, and superheating temperature = 800°C (Figure 11B). These maps emphasize a key distinction between morphology selection and morphology burden: the variables that decide which morphology forms need not be the same variables that control how much of the microstructure that morphology occupies once present.

In the Fe–Mn composition space (Figure 11A), the Chinese script exhibits an area‐fraction response dominated by Fe: the expected burden shifts from a low regime at Fe below ∼1.3 wt.% to an intermediate regime at higher Fe, while Mn has comparatively little influence within the formed domain. This decoupling is notable because Mn is a strong discriminator for Chinese‐script formation (Section 2.3), yet once the script morphology is stabilized, Fe becomes the primary lever controlling its areal burden. Polyhedral morphology shows the opposite tendency: its expected area fraction increases strongly with Mn and reaches a high regime at Mn ≳ 1.5–2.0 wt.% with weak Fe sensitivity, indicating that Mn amplifies both selection and burden for the polyhedral field. Coarse platelets display an inverse gradient, with areal burden increasing with Fe and decreasing with Mn, consistent with Mn mitigating platelet severity even in Fe‐rich compositions. Fine platelets, within their limited formed region on this slice, remain largely in an intermediate area‐fraction regime, suggesting that their distinguishing severity signatures are expressed more strongly through population and geometry descriptors than through areal occupancy alone.

The Fe–cooling‐rate slice (Figure 11B) highlights kinetic redistribution at fixed Mn and Si. Chinese script carries higher area fraction at low cooling rates (remaining in an intermediate regime up to ∼20°C s^−1^) and declines as cooling increases, consistent with slower solidification enabling greater growth/coarsening of script‐like features, as commonly observed in cast Al–Si alloys containing Fe‐rich intermetallics [38]. Polyhedral morphology shows the opposite trend: its expected area fraction increases with cooling rate and reaches a high regime above ∼40°C s^−1^, indicating that faster cooling shifts both morphology preference and areal burden toward polyhedral Fe‐IMCs under this composition anchor, in agreement with prior observations that rapid solidification favors refinement and compactification of Fe‐containing intermetallic populations [39]. Coarse platelets exhibit a non‐monotonic dependence with a peak near moderate cooling (∼10°C s^−1^), suggesting a kinetic “sweet spot” for platelet development rather than a single‐direction refinement effect. Fine platelets are not supported on this slice under the formation‐probability masking criterion, consistent with their suppression at Mn = 1 wt.% in Section 2.3.

**FIGURE 12:**
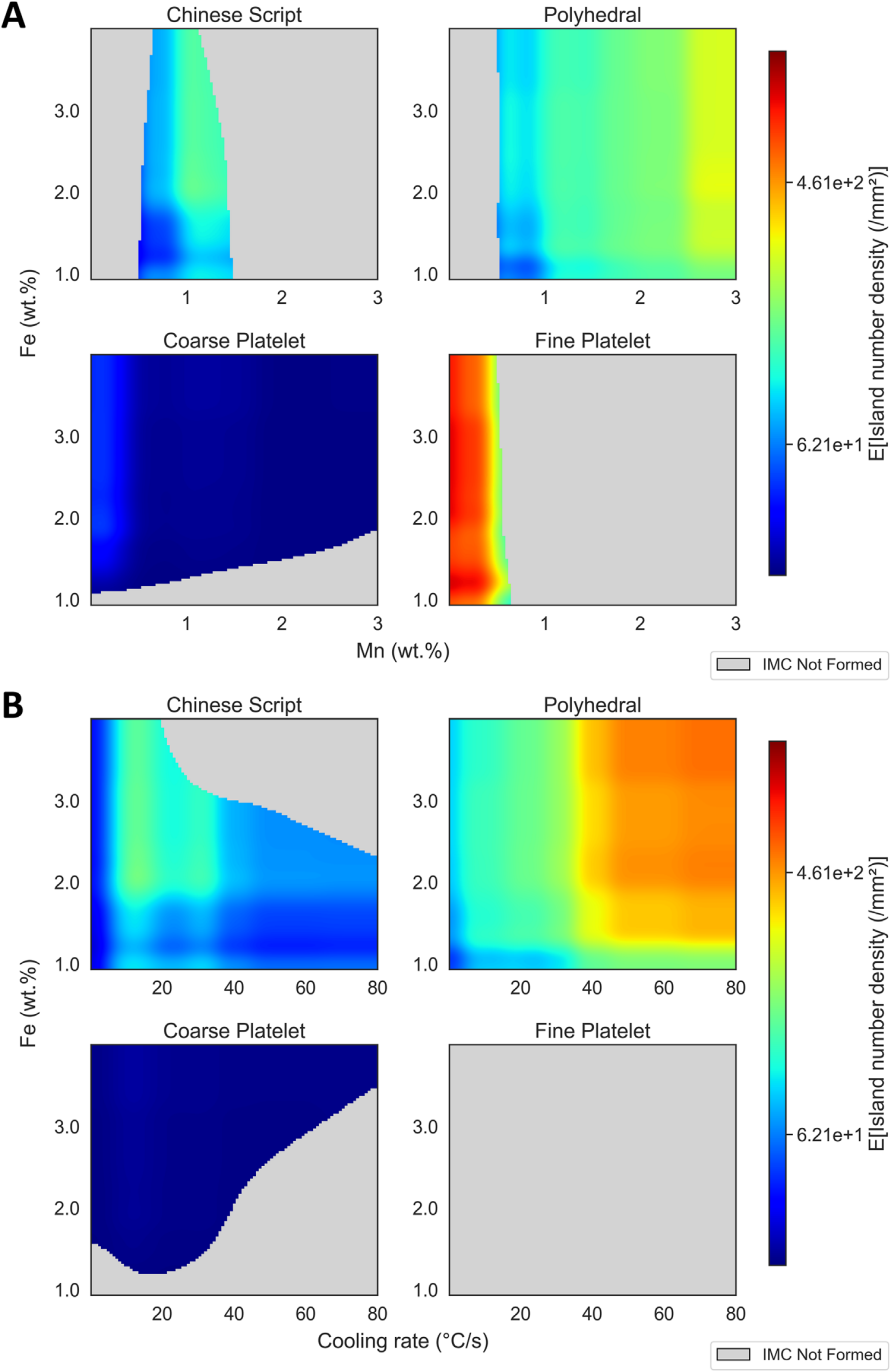
Composition–property maps showing the expected island number density of Fe‐rich intermetallic compounds (Fe‐IMCs), $E[\mathrm{Island}\;\mathrm{number}\;\mathrm{density}\;(mm^{-2})]$, predicted by the ordinal cumulative models. (A) Fe and Mn contents at fixed Si = 11 wt.%, cooling rate = 10°C s^−1^, and superheating temperature = 800°C. (B) Fe content and cooling rate at fixed Si = 11 wt.%, Mn = 1 wt.%, and superheating temperature = 800°C. Separate panels correspond to Chinese script, polyhedral, coarse platelet, and fine platelet morphologies. Color represents the expected ordinal response level, obtained as the probability‐weighted expectation of the predicted bins, while grey regions indicate conditions where the corresponding morphology is not expected to form based on the GAMI‐Net formation probability (probability < 0.5).

The total area fraction captures how much of the microstructure is occupied, but not whether that burden is carried by many small features or few dominant ones. Figure 12 therefore reports expected island number density regimes, providing a complementary population‐level signature.

In the Fe–Mn composition space (Figure 12A), the Chinese script transitions from low to intermediate number density values as Fe and Mn increase within the formed region, indicating that in some composition windows script burden is expressed through both increased coverage and a more populous feature network. Polyhedral morphology shows clearer stratification with Mn, progressing from low density at low Mn to intermediate density over mid‐Mn and reaching high density at Mn ≳ 2.5 wt.%. Combined with the area‐fraction map, this defines a high‐Mn polyhedral regime characterized by both extensive coverage and dense feature populations. Coarse platelets remain in a low density regime across most of their formed domain even where area fraction rises, implying that coarse platelet burden is typically carried by a small number of prominent features rather than by a finely dispersed population. Fine platelets, where supported, exhibit higher number density at very low Mn and shift toward intermediate density approaching the upper limit of their formation window, consistent with fine platelets being expressed through more numerous features than coarse platelets when accessible.

In Fe–cooling‐rate space (Figure 12B), Chinese script shows a band of intermediate number density at moderate cooling (∼10–40°C s^−1^), with lower densities at both slow and fast extremes. Notably, this population maximum does not coincide with the highest Chinese‐script area fraction (which occurs at low cooling), implying a redistribution from growth‐dominated burden at slow cooling to population‐dominated burden at moderate cooling. Polyhedral number density increases strongly with cooling rate and reaches a high regime above ∼40°C s^−1^, reinforcing that the high‐cooling polyhedral field is both more extensive and more populous. Coarse platelet density remains low across the plane, again supporting a “few‐feature” burden signature. Fine platelets are not supported on this slice under the masking criterion, consistent with their low formation probability at Mn = 1 wt.% in Section 2.3.

**FIGURE 13:**
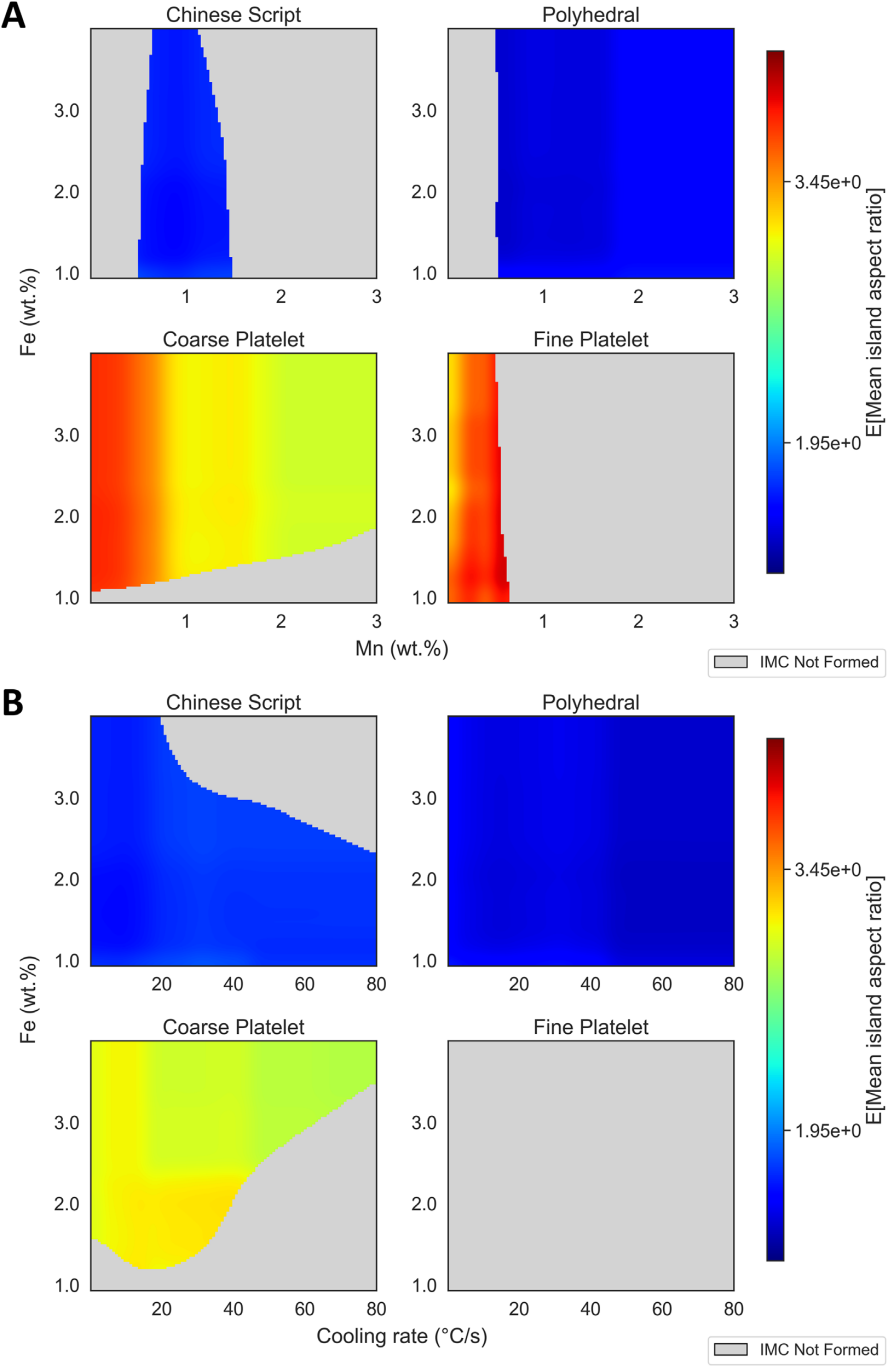
Composition–property maps showing the expected mean island aspect ratio of Fe‐rich intermetallic compounds (Fe‐IMCs), E[Meanislandaspectratio], predicted by the ordinal cumulative models. (A) Fe and Mn contents at fixed Si = 11 wt.%, cooling rate = 10°C s^−1^, and superheating temperature = 800°C. (B) Fe content and cooling rate at fixed Si = 11 wt.%, Mn = 1 wt.%, and superheating temperature = 800°C. Separate panels correspond to Chinese script, polyhedral, coarse platelet, and fine platelet morphologies. Color represents the expected ordinal response level, obtained as the probability‐weighted expectation of the predicted bins, while grey regions indicate conditions where the corresponding morphology is not expected to form based on the GAMI‐Net formation probability (probability < 0.5).

Figure 13 maps expected mean island aspect ratio regimes and provides an internal consistency check: compact morphologies should remain in low‐aspect‐ratio regimes, whereas platelet‐like morphologies should occupy higher‐aspect‐ratio regimes. This expected separation is clearly preserved. Chinese script and polyhedral morphologies remain confined to low aspect ratio across their formed domains (Figure 13A,B), indicating that composition and processing predominantly redistribute burden via extent, population, and spacing rather than altering intrinsic compactness. Coarse platelets occupy intermediate‐to‐high aspect ratio regimes and show a clear Mn dependence in Fe–Mn space: at Mn ≲ 1 wt.% platelets are most elongated, while increasing Mn shifts the expected response toward lower aspect ratios. Importantly, Mn thus moderates platelet severity not only by reducing formation likelihood and areal burden, but also by reducing elongation when platelets form. Fine platelets, where supported in Fe–Mn space, occupy the highest aspect‐ratio regime with weak Fe dependence, underscoring that this class is defined primarily by geometry once accessed.

**FIGURE 14:**
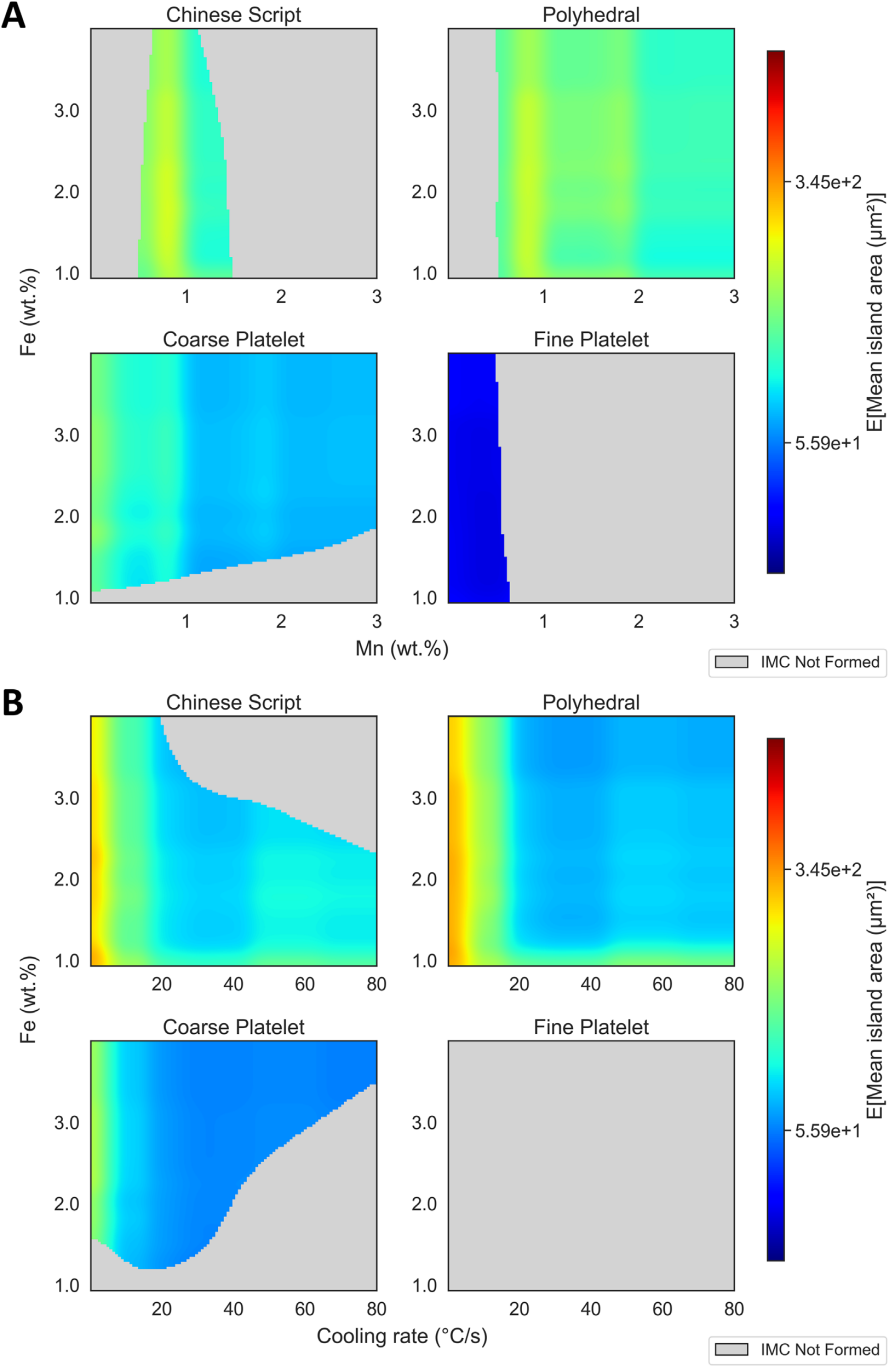
Composition–property maps showing the expected mean island area of Fe‐rich intermetallic compounds (Fe‐IMCs), $E[\mathrm{Mean}\;\mathrm{island}\;\mathrm{area}\;(\mu m^{2})]$, predicted by the ordinal cumulative models. (A) Fe and Mn contents at fixed Si = 11 wt.%, cooling rate = 10°C s^−1^, and superheating temperature = 800°C. (B) Fe content and cooling rate at fixed Si = 11 wt.%, Mn = 1 wt.%, and superheating temperature = 800°C. Separate panels correspond to Chinese script, polyhedral, coarse platelet, and fine platelet morphologies. Color represents the expected ordinal response level, obtained as the probability‐weighted expectation of the predicted bins, while grey regions indicate conditions where the corresponding morphology is not expected to form based on the GAMI‐Net formation probability (probability < 0.5).

Figure 14 maps expected mean island area regimes and, when interpreted alongside number density (Figure 12), clarifies whether burden changes arise from growth of fewer large features or proliferation of smaller ones. In Fe–Mn space (Figure 14A), Chinese script and polyhedral morphologies occupy predominantly intermediate mean‐area regimes with only modest local enhancements, whereas coarse platelet mean area decreases with increasing Mn, consistent with Mn jointly reducing platelet elongation (Figure 13A) and characteristic feature size, which reproduces the widely reported role of Mn in mitigating the severity of platelet‐like Fe‐rich intermetallics [24]. Fine platelets remain in the lowest mean‐area regime where supported, indicating a distinct structural state characterized by intrinsically small features rather than simply an underdeveloped coarse platelet.

In Fe–cooling‐rate space (Figure 14B), Chinese script and polyhedral mean island area decreases with increasing cooling rate, consistent with kinetic refinement reducing characteristic feature size. When combined with the strong increase in polyhedral number density with cooling (Figure 12B), the polyhedral field exhibits a coherent refinement signature: more numerous, smaller compact intermetallic features at higher cooling rates. Chinese script shows a similar size reduction with cooling, but its density peaks at intermediate cooling rather than increasing monotonically; together with the decline in area fraction at high cooling (Figure 11B), this indicates that fast solidification suppresses script burden through both limited growth and, beyond a threshold, reduced population development. Coarse platelet mean area also decreases with cooling, but because platelet density remains low, variations in areal burden at moderate cooling are more consistent with changes in feature development and growth kinetics than with population multiplication, highlighting a fundamental mechanistic difference between compact and platelet‐like morphologies.

**FIGURE 15:**
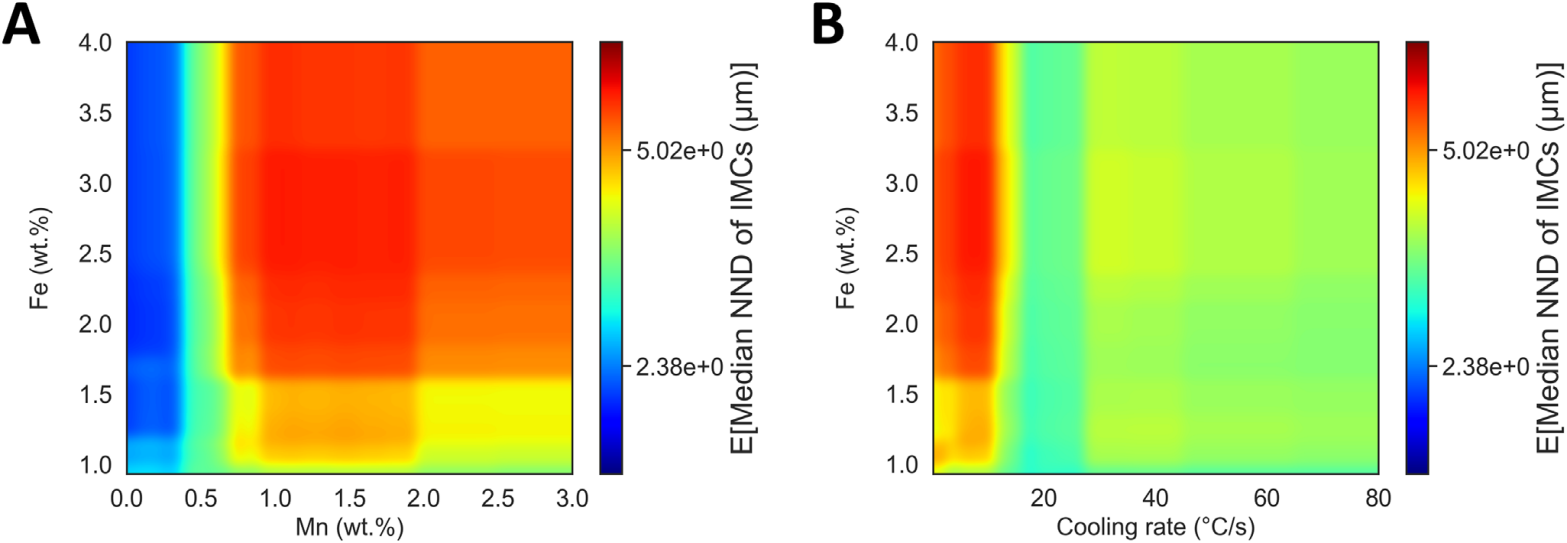
Composition–property maps showing the expected median nearest‐neighbor distance (NND) of Fe‐rich intermetallic compounds (IMCs), E[MedianNNDofIMCs(μm)], predicted by the ordinal cumulative models. (A) Fe and Mn contents at fixed Si = 11 wt.%, cooling rate = 10°C s^−1^, and superheating temperature = 800°C. (B) Fe content and cooling rate at fixed Si = 11 wt.%, Mn = 1 wt.%, and superheating temperature = 800°C. Color represents the expected ordinal response level, obtained as the probability‐weighted expectation of the predicted bins.

Figure 15 reports the expected median nearest‐neighbor distance (NND), an integrated descriptor reflecting both population density and spatial dispersion. In Fe–Mn space (Figure 15A), low Mn compositions correspond to low spacing (low NND) across all Fe content ranges, while NND rises sharply as Mn enters intermediate regimes and reaches large values at higher Fe content. This nonlinear response indicates that Mn reorganizes spatial structure rather than simply shifting spacing uniformly. In the Fe–cooling‐rate space (Figure 15B), cooling rate drives a pronounced transition: low cooling rates produce large spacing across the entire Fe range, whereas increasing cooling into moderate regimes sharply reduces spacing, and high cooling rates yield comparatively uniform NND with weak Fe sensitivity. In combination with Figures 11, 12, 13, 14, this behavior is consistent with cooling rate acting as a dominant control parameter for spatial refinement, compressing characteristic spacing and reducing compositional sensitivity within the explored domain.

In summary, the ordinal model–derived composition–property landscapes provide a morphology‐resolved and quantitative view of how composition and processing redistribute Fe‐intermetallic burden beyond simple presence or absence. When interpreted together with the morphology probability maps (Section 2.3) and pairwise driver landscapes (Section 2.4), these regime maps reveal that effective microstructural control requires managing both morphology selection and burden partitioning within a morphology. Compact morphologies (Chinese script and polyhedral) consistently exhibit low aspect ratios, yet they shift between growth‐dominated and population‐dominated burden states depending on kinetics. Platelet‐like morphologies concentrate the contaminant‐related microstructure burden into fewer, more elongated features and display distinct sensitivities to chemistry and thermal history, with the Mn content acting as an important stabilizing lever that can reduce both platelet likelihood and platelet severity when present. Together, these morphology‐conditioned landscapes complete the Results by linking selection, driver attribution, and burden partitioning into a coherent set of actionable maps, providing the quantitative foundation for the mechanistic discussion that follows.

## 3 Discussion

The key message of this study is that controlling Fe‐IMCs in recycled Al cast alloys is not a binary problem of “present versus suppressed.” It is instead a continuous design problem in which the unavoidable Fe burden is allocated within microstructural space. That burden may be concentrated into a small number of elongated, crack‐prone features, redistributed into a dense population of compact particles, or expressed in intermediate regimes characterized by distinct size and spacing signatures. Reframing the problem this way changes how composition and processing should be used in practice. Instead of treating intermetallic control as a single lever aimed at eliminating “bad phases,” it becomes a two‐stage design task: first selecting the morphology field that is most mechanically tolerable, and then tuning how severe that morphology becomes once it forms.

This two‐stage framing is the central novelty of the present study. The Results show that “morphology selection” and “burden partitioning” are related but not interchangeable questions: variables that decide which morphology appears are not necessarily the same variables that determine how much microstructural burden that morphology carries and how that burden is organized (coverage, population, elongation, characteristic size, and spacing). This distinction matters because two alloys can exhibit similar overall intermetallic fraction yet differ fundamentally in damage susceptibility if the burden is carried by sparse platelets versus a refined compact population. By making this separation explicit and quantifiable, the framework provides a design logic that aligns closely with how metallurgists reason. On one hand, it reflects phase‐field‐like selection, and on the other, it captures severity and topology control, while remaining usable with sparse, industrially realistic datasets.

A second conceptual advance is the deliberate coupling of interpretability with prediction targets that are conservative by construction. On the selection side, GAMI‐Net replaces an opaque multivariate predictor with an additive structure that decomposes decisions into main effects and pairwise interactions. For readers less familiar with interpretable machine learning, this structure can be viewed as a data‐driven analogue of a response‐surface model in which single‐variable contributions and physically meaningful pairwise couplings are made explicit. The pairwise effect landscapes extend interpretability beyond fixed‐slice probability maps by isolating the net contribution of a chosen feature pair while marginalizing over all others. This addresses a common limitation of traditional parametric studies: trends observed at fixed conditions can obscure the true controlling interactions, whereas the marginalized pairwise landscapes reveal which couplings consistently govern morphology transitions across the broader sampled design space. In practical terms, this produces an interpretable hierarchy of “what controls what”: some morphologies are dominated by compositional windows, others respond strongly to kinetic levers, and still others reflect antagonistic tradeoffs such as Fe‐promoted platelet formation counteracted by Mn.

To further assess the physical credibility of these learned pairwise effects, we examine their consistency with established metallurgical knowledge derived from phase diagrams and solidification kinetics. Several dominant trends recovered by the model align closely with classical understanding. For example, the Mn‐driven suppression of platelet‐like Fe‐intermetallics and the promotion of compact α‐type morphologies are well documented in Al–Si–Fe–Mn systems, where Mn modifies phase stability and shifts the *β→α* transformation pathway during solidification. The antagonistic Fe–Mn coupling identified in the pairwise landscapes is likewise consistent with the known role of Mn in compensating the detrimental effects of excess Fe. Similarly, the predicted influence of superheating temperature on promoting polyhedral morphologies agrees with metallurgical interpretations in which higher superheat alters nucleation conditions and reduces the persistence of pre‐existing intermetallic nuclei.

Importantly, no dominant pairwise effects were identified that clearly contradict established physical metallurgy principles within the explored domain. Minor variations or weak dependencies are more plausibly attributed to dataset limitations or the restriction to pairwise interactions, rather than to fundamentally new or conflicting mechanisms. In this sense, the model primarily recovers physically meaningful trends while providing a quantitative and continuous representation of their interaction structure across composition–processing space.

On the descriptor side, the use of ordinal cumulative models represents a complementary form of epistemic discipline. Instead of training regression models that encourage overconfident point predictions from limited data, the ordinal formulation treats descriptors as ordered regimes. Conceptually, this resembles classifying microstructures into acceptable, marginal, and critical ranges rather than predicting exact numerical values that may not be robust to sampling, segmentation, or sectioning variability. Reporting descriptor maps as expected‐bin surfaces yields smooth composition–property landscapes while preserving the ordinal meaning of transitions between regimes. This is particularly important for descriptors that are zero‐inflated or inherently intermittent, where “absence” is a physically distinct state from “small but finite.” In this sense, the modeling choices are not merely technical. They embody the design philosophy of the work, in which actionable regimes and interpretable transitions are prioritized over fragile numerical precision.

A further innovation is the way these components are connected into a coherent pipeline through morphology‐conditioned mapping and formation‐probability masking. Microstructural descriptors are only meaningful when the corresponding morphology exists, as such descriptors are inherently defined with respect to morphology‐specific features and mechanisms (e.g., grain size, dendrite arm spacing, or intermetallic morphology) [24, 29, 38]. Yet, many predictive workflows implicitly extrapolate descriptors into regions where the structure is not physically realized. By using the morphology selection model to define the feasible domain for descriptor interpretation, the framework avoids spurious “severity maps” in non‐formation regions and maintains physical consistency between selection and burden landscapes. This integration turns what could otherwise be disconnected models into a single, mechanistically legible design tool. Selection defines the admissible field, pairwise effects explain why that field shifts, and ordinal maps quantify how the burden evolves within it.

While the present work focuses on composition–processing–structure (CPS) relationships, the predicted morphology regimes and descriptor levels can be directly linked to mechanical performance through established metallurgical mechanisms. Previous studies have shown that platelet‐like Fe‐intermetallics act as strong stress concentrators, promoting early crack initiation and significantly reducing ductility and fatigue resistance, whereas more compact morphologies such as polyhedral and Chinese‐script forms lead to more homogeneous stress distribution and improved mechanical tolerance [14, 15, 17].

In the present framework, these effects can be interpreted through the predicted descriptor regimes. For example, coarse platelet morphologies correspond to high aspect ratio and low number density, representing a regime dominated by a small number of critical defects that can trigger localized fracture. In contrast, polyhedral morphologies formed under higher cooling rates or increased Mn content are associated with reduced feature size and more distributed populations, corresponding to a more damage‐tolerant microstructure state.

These connections indicate that the proposed morphology‐conditioned descriptors provide physically meaningful proxies for microstructure‐sensitive damage mechanisms. Although a direct quantitative prediction of mechanical properties is beyond the scope of the present study, the results suggest that the framework can be naturally extended toward composition–processing–structure–property (CPSP) linkages by using the predicted morphology probabilities and descriptor regimes as intermediate variables for property prediction.

From an industrial perspective, these outcomes address the core challenge of aluminum recycling: Fe is an unavoidable and variable contaminant associated with increasing scrap utilization, yet production decisions must be made quickly under tight cost and quality constraints. The model‐derived probability maps can support charge blending and alloying strategies by identifying composition windows that reduce the likelihood of mechanically harmful morphologies under representative casting conditions, and by enabling rapid adjustments when Fe levels vary between different scrap charges. The pairwise driver landscapes can prioritize the most impactful levers for a given foundry route, whether those involve chemistry coupling such as Fe–Mn balance, kinetics such as cooling rate, or melt thermal history such as superheating. This prioritization helps reduce trial‐and‐error iterations. The ordinal composition–property landscapes add a second layer of actionable guidance by indicating how processing adjustments can shift a feasible morphology into less severe burden regimes. Because inference is lightweight, the approach is compatible with integration into process monitoring systems and digital‐twin workflows where chemistry and thermal histories are tracked continuously.

Interpretability is also operationally significant for adoption. In industrial settings, “black‐box” outputs are often less actionable than explanations that can be reconciled with metallurgical intuition and used to justify process changes. The explicit exposure of dominant pairwise drivers and the regime‐based descriptor outputs provide traceable reasoning that supports decision‐making across multidisciplinary teams. This transparency also enables structured updates: when a new melt source or casting line introduces a systematic shift, the driver hierarchy offers a way to diagnose whether the deviation is rooted in chemistry coupling, cooling history, or melt thermal history, guiding targeted data collection rather than indiscriminate retraining.

Several limitations should be acknowledged. The dataset is modest and bounded; therefore, the maps should be interpreted primarily as interpolation within the sampled, industrially actionable domain, rather than as universal metallurgical rules, as further indicated by the nonlinearities observed. Accordingly, extrapolation to compositions or processing conditions outside the trained ranges (e.g., substantially lower Fe or Mn contents, or extreme cooling rates) should be treated with caution, as the learned relationships are not constrained by data in those regions.

In particular, the present dataset is representative of recycled Al–Si alloys, which typically contain Fe levels above ∼1 wt.%. For primary alloys with significantly lower Fe and Mn contents, thermodynamic considerations suggest that both the fraction and characteristic size of Fe‐containing intermetallic compounds would be reduced at a given cooling rate. Therefore, extrapolation of the present model to such low‐impurity regimes should be guided by physical reasoning and, where possible, additional data.

The GAMI‐Net structure captures univariate and pairwise interactions; while these are often the dominant effects in practice, this design choice also imposes a limitation, as higher‐order (ternary or beyond) couplings are not explicitly represented. Such interactions may become significant in complex industrial scenarios where multiple alloying elements and processing variables co‐vary, potentially leading to nonlinear effects that cannot be fully captured within the present pairwise framework.

Morphology labels and descriptors are derived from image‐based characterization and therefore inherit uncertainty from two‐dimensional sectioning, segmentation choices, and finite sampling. Finally, the study focuses on composition–processing–microstructure linkages; mechanical properties were not modeled directly, and property outcomes can be influenced by additional factors such as porosity, eutectic modification, oxide films, and defect populations.

These limitations point to clear future directions. Dataset expansion can be made efficient through active learning that targets experiments near morphology boundaries or descriptor transition corridors where uncertainty is highest. Incorporating physics‐informed features from thermodynamic calculations or solidification simulations could regularize behavior near domain edges and improve extrapolation safety. Extending the interpretable model class to allow structured higher‐order interactions would test when ternary couplings materially alter morphology selection. On the descriptor side, adding connectivity/topology metrics, 3D morphology measures, and phase‐resolved identification would strengthen the link to crack initiation, feeding behavior, and defect sensitivity. Finally, coupling the present microstructure regime maps to property models and multi‐objective optimization would close the design loop, enabling direct recommendation of composition–processing pathways that balance castability, microstructural risk, and performance under recycling‐driven variability.

In closing, this work shows that sparse yet carefully structured data can yield actionable microstructure design guidance when the modeling targets reflect how metallurgists reason about regimes, transitions, and tradeoffs. By unifying morphology selection, interpretable driver attribution, and morphology‐conditioned burden partitioning, the proposed framework moves Fe‐intermetallic control from qualitative rules toward explainable, optimization‐ready design maps. More broadly, it illustrates a practical pathway for turning recycling variability, which has traditionally been a barrier, into a controllable design dimension in low‐carbon aluminum manufacturing.

## 4 Methods

### 4.1 Data Preparation

The dataset of Fe‐IMCs descriptors (area fraction, size, aspect ratio, area, circularity, solidity, number density, branch thickness, and nearest‐neighbor distance) corresponding to specific combinations of alloy composition and processing parameters (Si wt.%, Fe wt.%, Mn wt.%, cooling rate, and melt superheating temperature) was constructed using image analysis of scanning electron microscopy and optical microscopy micrographs obtained from the published literature. In total, 71 micrographs were collected from 23 different literature sources [17, 18, 19, 20, 21, 22, 23, 25, 26, 35, 40, 41, 42, 43, 44, 45, 46, 47, 48, 49, 50, 51, 52].

The workflow began with screening the literature for suitable microstructure images. Only micrographs that satisfy three compositional criteria were selected for further analysis. These criteria were designed to confine the dataset to the compositional tolerance of recycled Al–Si–Fe–Mn alloys without considerable modification of the as‐cast microstructure from grain refiners, eutectic modifiers, or additional transition metals (e.g., Cr), in order to minimize noise in the data.

First, the alloys corresponding to the selected micrographs must contain more than 1 wt.% Fe and 6 wt.% Si, which are the typical values for recycled Al–Si–Fe–Mn cast alloys. Second, because this study focuses on Mn as the primary transition metal used to mitigate the deleterious effects of platelet Fe‐IMCs, the Cr content was restricted to below 0.1 wt.% to avoid noise from Cr‐induced modification of Fe‐IMCs. Third, the maximum concentrations of common grain refiners and eutectic modifiers across all data points were limited to 270 ppm Sr, 40 ppm B, and 0.16 wt.% Ti, thereby eliminating any associated noise.

For each selected micrograph, the corresponding alloy composition, cooling rate, and melt superheating temperature were recorded. Whenever the cooling rate is not explicitly reported, it was estimated from the Secondary Dendrite Arm Spacing (SDAS) using empirical relationships [47, 53, 54, 55, 56, 57]. Because the relationship between SDAS and cooling rate in Al–Si alloys depends strongly on the Si content, the most appropriate published empirical relationship was selected for each alloy according to its specific composition. This composition‐specific approach was adopted to minimize the uncertainty in the cooling‐rate estimates. Once the micrographs and their associated composition and processing parameters were compiled, statistical descriptors of the Fe‐IMCs were calculated using ImageJ/Fiji software [58].

Because the micrographs were collected from different literature sources, their image quality varied, including differences in resolution, contrast, and imaging modality (optical microscopy vs. scanning electron microscopy). Such heterogeneity can introduce systematic variability in extracted descriptors, particularly for features sensitive to image thresholding and segmentation, such as area fraction, branch thickness, and circularity. To mitigate these effects, the image cleaning and segmentation procedures were adjusted on a micrograph‐by‐micrograph basis. Image sharpness and contrast were enhanced where necessary to enable reliable phase discrimination, and segmentation‐related noise was reduced using ImageJ filters such as “Despeckle” and “Remove Outliers” [58]. In cases where intermetallic particles were in contact or partially overlapping, segmentation was refined using threshold adjustment and watershed‐based separation to avoid artificial merging of adjacent intermetallic features. These steps were applied individually to ensure that each segmented binary image accurately represented the Fe‐IMC phases in the original micrograph. Representative examples of segmented binary micrographs are shown in Figure 4. While some level of measurement uncertainty due to inter‐source variability cannot be completely eliminated, the tailored preprocessing substantially reduces systematic bias. In addition, the use of ordinal cumulative models (low/intermediate/high regimes) inherently absorbs residual uncertainty by focusing on robust regime‐level distinctions rather than precise numerical values, which are more sensitive to image quality variations. This modeling choice therefore reduces the sensitivity of the framework to inter‐source variability and enhances the reliability of the extracted trends.

The Fe‐IMCs were classified into four distinct morphological categories: coarse platelet, fine platelet, Chinese‐script, and polyhedral. This classification is based solely on morphology and does not distinguish between differences in chemical composition or crystal structure. All Fe‐IMCs sharing the same morphology are grouped into a single category. The distinction between coarse and fine platelet morphologies is based on their formation behavior. Fine platelets are embedded within the interdendritic regions, indicating that they form at a later solidification stage through eutectic reactions with Al dendrites. In contrast, coarse platelets are not confined to interdendritic regions, suggesting that they form as primary crystals at the onset of solidification prior to Al dendrite formation.

For all four morphology categories, we calculated the following descriptors: area fraction (%), mean and maximum Feret diameter (µm), mean and maximum particle area (µm^2^), mean and maximum aspect ratio, and number density (particles/µm^2^).

Additional descriptors were calculated for the Chinese‐script and polyhedral morphologies due to their increased geometric complexity compared to the simple platelet shape. For the Chinese‐script morphology, branch thickness (µm) and circularity were included. Branch thickness represents the mean thickness of the script branches. Circularity is defined as 4π×Area/Perimeter^2^ [59]. Circularity was calculated because two Chinese‐script Fe‐IMCs may have identical bounding width and length dimensions, hence equal aspect ratios. Nevertheless, they may differ significantly in branching density, growth pattern, and perimeter complexity. Highly branched script‐like particles with thin branches and numerous concavities tend to exhibit low circularity.

For polyhedral Fe‐IMCs, solidity was calculated in addition to circularity and branch thickness. Solidity is defined as the ratio of the particle area to its convex hull area [59]. As discussed earlier, polyhedral Fe‐IMCs may appear as solid or hollow rhombic particles or as star‐like branched structures. Circularity alone cannot fully distinguish between these morphologies. Higher solidity values indicate predominantly solid polyhedral particles, while lower values correspond to increasingly branched star‐like morphologies. Solidity was not calculated for Chinese‐script particles, as they are inherently branched.

Finally, the median nearest‐neighbor distance (µm) between Fe‐IMCs was calculated without segmentation between the morphology categories. The edge‐to‐edge nearest‐neighbor distance between an Fe‐IMC particle and its neighboring particles that share a Voronoi cell boundary was determined. Then, the overall median value was calculated. The median was used instead of the mean because the distribution of nearest‐neighbor distances was skewed. A spreadsheet containing the complete dataset is provided in the supporting information.

### 4.2 Machine Learning Models

To predict whether a given Fe rich intermetallic compound morphology forms under a specified combination of alloy composition and processing conditions, we developed an interpretable neural network classifier based on a generalized additive model with pairwise interactions, hereafter referred to as a GAMI style network [27, 28]. The complete modeling workflow was implemented in Python using the PyTorch framework [60].

Let $x\in R^{5}$ denote the standardized input vector comprising Si (wt.%), Fe (wt.%), Mn (wt.%), cooling rate (°C s^−1^), and superheating temperature (°C). For a binary outcome indicating whether a given morphology forms, the model produces a logit $\hat{y}$, which is converted to a probability through a sigmoid activation. The logit is expressed as an additive decomposition of univariate main effects and pairwise interaction effects,

(7) $$\hat{y}=\beta_{0}+\sum_{i=1}^{d}f_{i}\left(x_{i}\right)+\sum_{1\leq i<j\leq d}f_{i,j}\left(x_{i},x_{j}\right),\;d=5$$

where β_0_ is a learned bias term, *f_i_* (·) represents the contribution of an individual feature, and *f*_*i*,*j*_(·, ·) captures the interaction between a pair of features. All ten possible pairwise interactions among the five input variables were included. This explicit additive structure enables direct interpretation of learned relationships through inspection of the individual main effect functions and interaction surfaces, without the need for post hoc explainability techniques.

Each main effect component *f_i_* was parameterized by a fully connected sub network with six linear layers arranged as 1–h–h–h–h–h–1, while each interaction component *f*_*i*,*j*_ used an analogous architecture with a two dimensional input. Gaussian Error Linear Unit activations were applied between layers, and the hidden layer width was set to (h = 4).

Because morphology formation was evaluated separately for each morphology category, namely Chinese script, polyhedral, coarse platelet, and fine platelet, a distinct binary classifier was trained for each morphology. After constructing a stratified hold out split, the data were grouped by morphology type, and an independent GAMI style model was trained for each category using the same five numeric input features.

All numeric inputs were standardized by z score normalization using statistics computed from the training partition only. The dataset was divided into training and test subsets using a fixed random seed with stratification by morphology type, with a 90 to 10 split. To improve robustness and reduce variance, an internal tenfold cross‐validation ensemble was trained within the training partition for each morphology category. For each fold, a separate model was trained on the fold specific training subset and evaluated on the corresponding validation subset. Training proceeded for up to 500 epochs using the Adam optimizer with a learning rate of 1 × 10^−3^, weight decay of 1 × 10^−5^, and a mini batch size of 8 [61]. Early stopping was applied based on validation loss with a patience of 40 epochs, and the checkpoint corresponding to the lowest validation loss was retained.

To stabilize optimization, label smoothing with a factor of 0.1 and gradient clipping with a maximum norm of 1.0 were applied. Class imbalance was addressed using a weighted binary cross‐entropy loss, with the positive class weight computed from the fold specific training labels. Final ensemble predictions were obtained by averaging predicted probabilities across the ten cross‐validation folds. During training, model behavior was monitored using binary cross‐entropy loss together with threshold dependent metrics such as accuracy at a decision threshold of 0.5.

Quantitative morphology descriptors were predicted using ordinal cumulative models trained with gradient boosted decision trees implemented in CatBoost [62]. The numeric input features were the same five composition and processing variables used for the GAMI style classifiers. For all descriptor datasets except nearest‐neighbor distance, the morphology category was additionally included as a categorical input feature. Samples containing missing values were removed prior to modeling.

Because many descriptor targets were strictly positive and strongly right skewed, continuous values were first transformed using log(1+y). The transformed targets were then discretized into three ordered bins representing low, intermediate, and high regimes. Bin boundaries were determined using a clustering based approach in which 1D k‐means clustering was applied to the transformed target distribution to form fine‐grained clusters, followed by selection of bin edges that produced approximately balanced sample counts. For zero‐inflated descriptors such as area fraction and island number density, all zero‐valued samples were assigned to the lowest bin, and the remaining positive values were discretized separately before merging.

For a target with *K* ordinal bins, *K* − 1 binary classifiers were trained to model exceedance probabilities *P*(*y* > *t*) for thresholds t∈{0,…,K−2}. Each threshold classifier was implemented using CatBoost with GPU acceleration and automatic class balancing. Hyperparameters were fixed across all targets and included a log‐loss objective, a maximum of 3000 boosting iterations, a learning rate of 0.015, and a tree depth of 8. Early stopping was applied based on validation performance with a patience of 200 iterations. When applicable, the column index of the categorical morphology feature was provided explicitly to CatBoost. Unlike the GAMI style networks, CatBoost models were trained on the original unstandardized feature scales.

For each descriptor target, the data were split once into training, validation, and test partitions with proportions of 0.8, 0.1, and 0.1 using a stratified strategy designed to avoid degenerate splits for rare labels. A single ordinal cumulative model was trained per descriptor target, without ensembling across random seeds or folds. At inference time, predicted exceedance probabilities were postprocessed to enforce monotonicity, such that (*P*(*y* > *t*) ≥ *P*(*y* > *t* + 1)). Per‐bin probabilities were then computed from differences of adjacent cumulative probabilities and normalized for numerical stability, and the predicted ordinal class was selected as the maximum probability bin.

Model performance was evaluated using overall accuracy together with per class precision, recall, and F1 score, and visualized using confusion matrices for both morphology classifiers and ordinal descriptor models. All trained models, binning metadata including bin edges and labels, and data splits were serialized to enable fully reproducible inference and figure generation. The source code used for model training, evaluation, and inference in this study is available in the Code Availability section [64]. The repository includes the full implementation, trained models, data splits, and preprocessing workflows required to reproduce all results and figures reported in this work.

To benchmark the necessity of a neural‐network‐based model for the present dataset size, we additionally evaluated several simpler baseline classifiers, including logistic regression with explicit pairwise interaction terms, random forest, and histogram‐based gradient boosting. All baseline models were trained using the same input features (Si, Fe, Mn, cooling rate, and superheating temperature) and the same train–test split as used for the GAMI‐Net models. For logistic regression, pairwise interaction terms between all input variables were included to ensure a fair comparison with the pairwise structure of GAMI‐Net. Tree‐based models were implemented using standard library defaults with minor adjustments to ensure stable training. Model performance was evaluated using the same metrics as for GAMI‐Net, including accuracy, precision, recall, F1 score, AUROC, and average precision.

## Author Contributions

J. Wang (Jaemin Wang): Conceptualization, Methodology, Software, Validation, Formal analysis, Investigation, Data curation, Writing – original draft, Writing – review & editing, Visualization, Project administration. W. Mohammed (Waleed Mohammed): Conceptualization, Methodology, Software, Validation, Formal analysis, Investigation, Data curation, Writing – original draft, Writing – review & editing, Visualization, Project administration. D. Raabe (Dierk Raabe): Conceptualization, Methodology, Funding acquisition, Project administration, Writing – review & editing

## Code Availability

The source code used for model training, evaluation, and inference in this study is available for download at https://doi.org/10.5281/zenodo.18059244.

## Conflicts of Interest

The authors declare no conflicts of interest.

## Supporting information




**Supporting file**: advs75446‐sup‐0001‐SuppMat.docx

**Supporting file 1**: advs75446‐sup‐0002‐ DataFile.xlsx

## Data Availability

The data that support the findings of this study are openly available in Zenodo at https://doi.org/10.5281/zenodo.18059244.
