# Supplementary material for: Data‐Driven Modeling of Composition–Processing–Microstructure Relations for Recycled Aluminum Cast Alloys
Source: Adv Sci (Weinh). 2026 Apr 30:e75446. Online ahead of print. doi: 10.1002/advs.75446 (PMC13335706; doi:10.1002/advs.75446)
Supplement: Supplementary file 1 — Supporting file: advs75446‐sup‐0001‐SuppMat.docx [file ADVS-9999-e75446-s001.docx]

**Supporting Information**

**Data-Driven Modeling of Composition–Processing–Microstructure Relations for Recycled Aluminum Cast Alloys**

Jaemin Wang^a,b,1,*^, Waleed Mohammed^a,1^, Dierk Raabe^a,*^

^a^ Max Planck Institute for Sustainable Materials, Düsseldorf 40237, Germany

^b^ Department of Materials Science and Metallurgical Engineering, Kyungpook National University, Daegu 41566, Republic of Korea

^1^ These authors contributed equally: Jaemin Wang, Waleed Mohammed

* Corresponding author (Jaemin Wang), E-mail: jmwang@knu.ac.kr

* Corresponding author (Dierk Raabe), E-mail: d.raabe@mpi-susmat.de

**Table S1.** Benchmark comparison of GAMI-Net and simpler baseline models for morphology classification.

| Model | Morphology | Test Accuracy (%) | Test Precision (%) | Test Recall (%) | Test F1 (%) |
| --- | --- | --- | --- | --- | --- |
| GAMI-Net | Chinese Script | 85.7 | 100 | 75 | 85.7 |
| Histogram-based gradient boosting |  | 85.7 | 100 | 75 | 85.7 |
| Logistic regression |  | 85.7 | 80 | 100 | 88.9 |
| Random forest |  | 85.7 | 100 | 75 | 85.7 |
| GAMI-Net | Polyhedral | 87.5 | 100 | 75 | 85.7 |
| Histogram-based gradient boosting |  | 87.5 | 100 | 75 | 85.7 |
| Logistic regression |  | 87.5 | 100 | 75 | 85.7 |
| Random forest |  | 87.5 | 100 | 75 | 85.7 |
| GAMI-Net | Coarse Platelet | 100 | 100 | 100 | 100 |
| Histogram-based gradient boosting |  | 71.4 | 0 | 0 | 0 |
| Logistic regression |  | 85.7 | 100 | 50 | 66.7 |
| Random forest |  | 85.7 | 100 | 50 | 66.7 |
| GAMI-Net | Fine Platelet | 100 | 100 | 100 | 100 |
| Histogram-based gradient boosting |  | 100 | 100 | 100 | 100 |
| Logistic regression |  | 85.7 | 100 | 75 | 85.7 |
| Random forest |  | 100 | 100 | 100 | 100 |

**
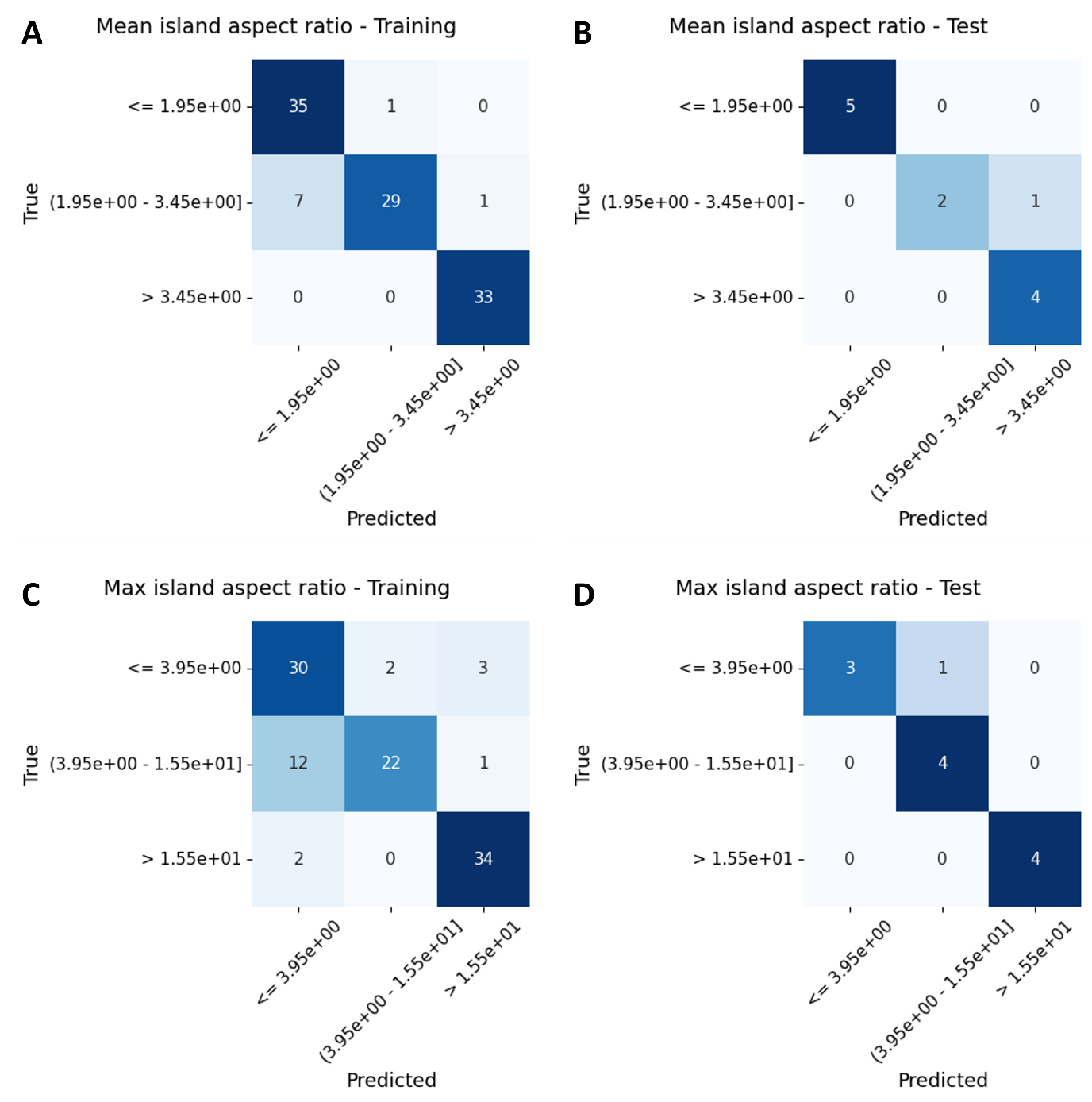
**

**Figure S1.** Confusion matrices for the ordinal cumulative model predicting (A,B) mean island aspect ratio and (C,D) max island aspect ratio. Panels (A,C) correspond to the training set, and (B,D) correspond to the test set.


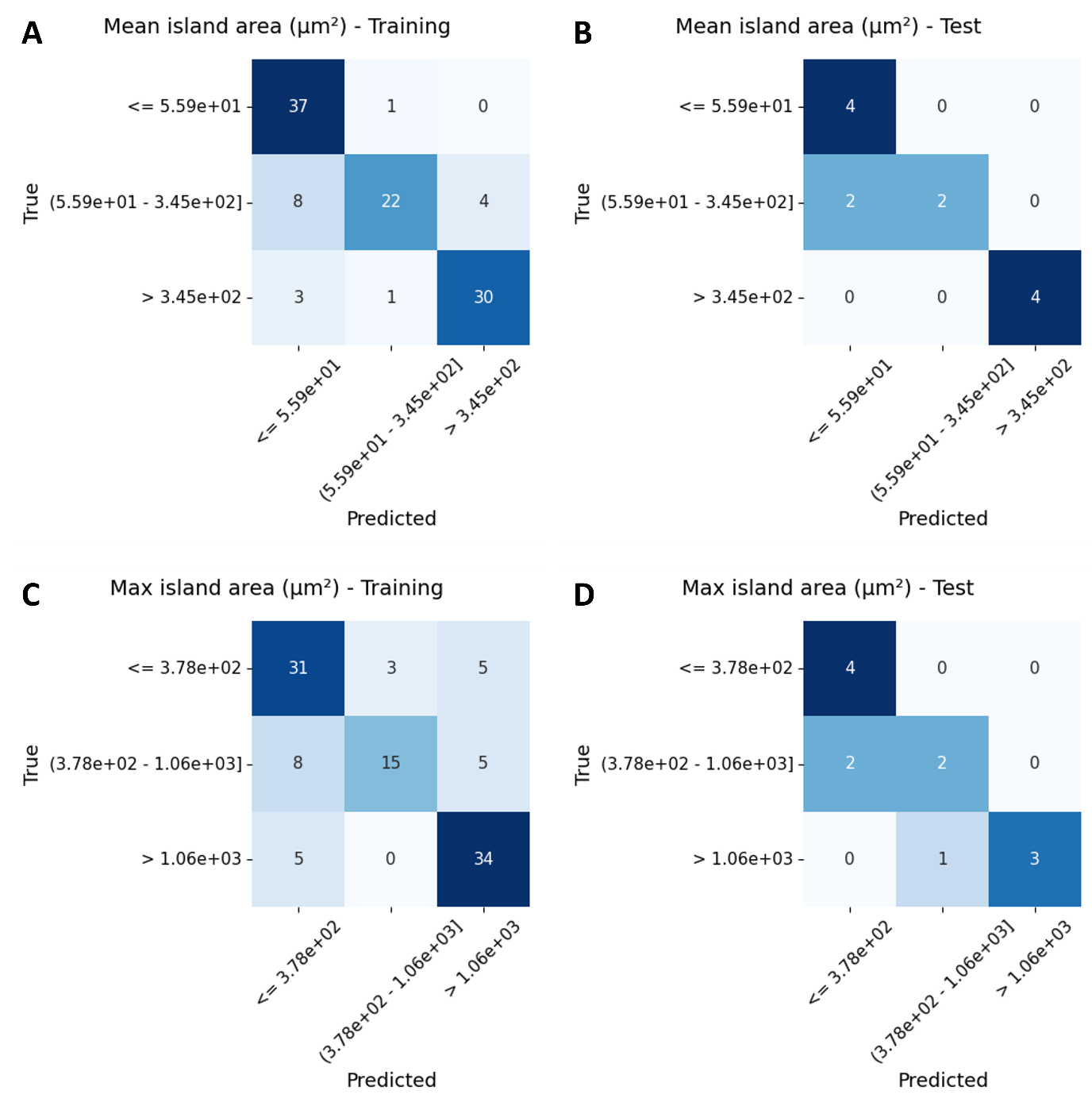


**Figure S2.** Confusion matrices for the ordinal cumulative model predicting (A,B) mean island area (μm²) and (C,D) max island area (μm²). Panels (A,C) show the training results, while (B,D) present the test performance.


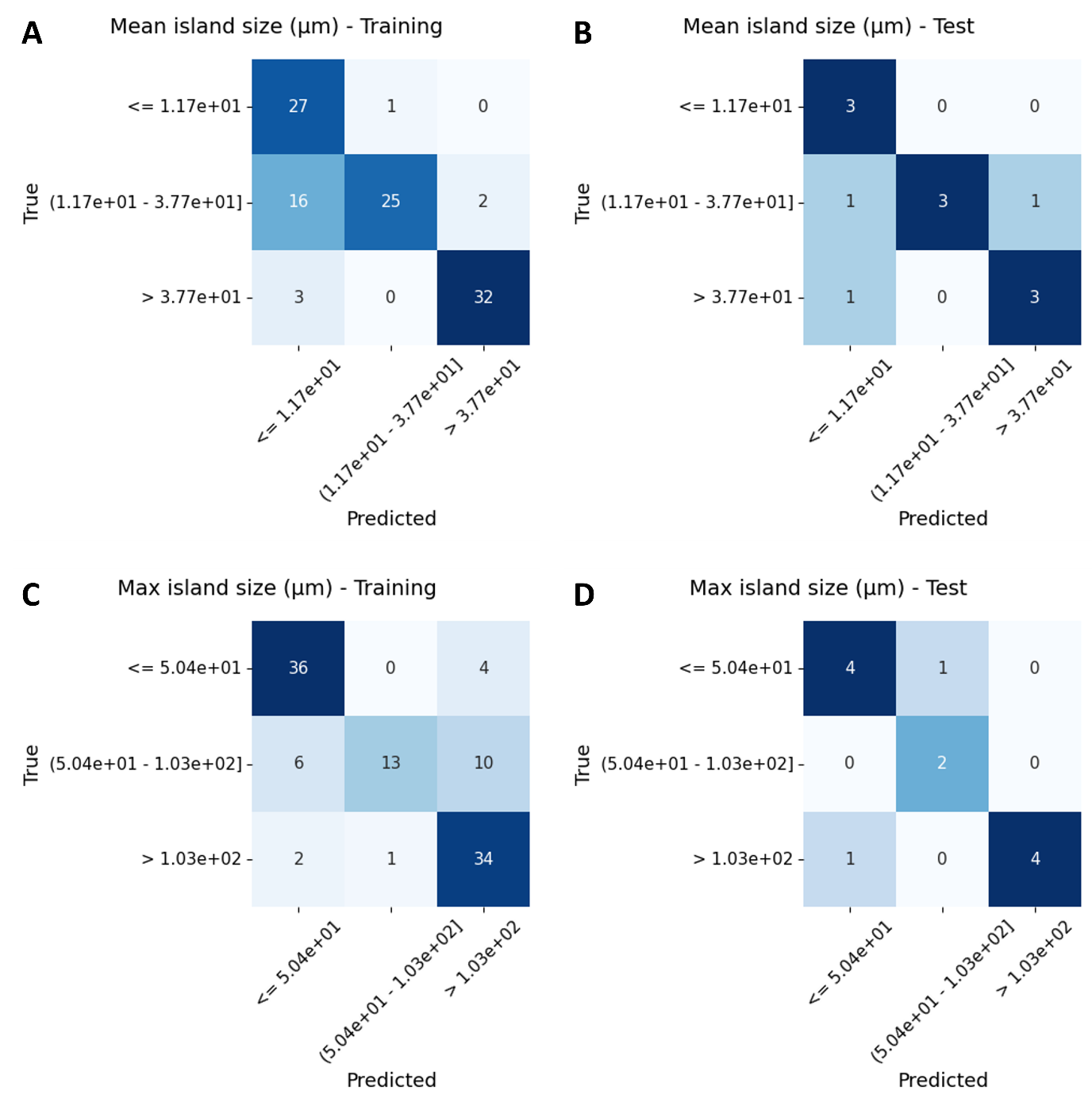


**Figure S3.** Confusion matrices for the ordinal cumulative model predicting (A,B) mean island size (μm) and (C,D) max island size (μm). Training and test sets are shown in (A,C) and (B,D), respectively.


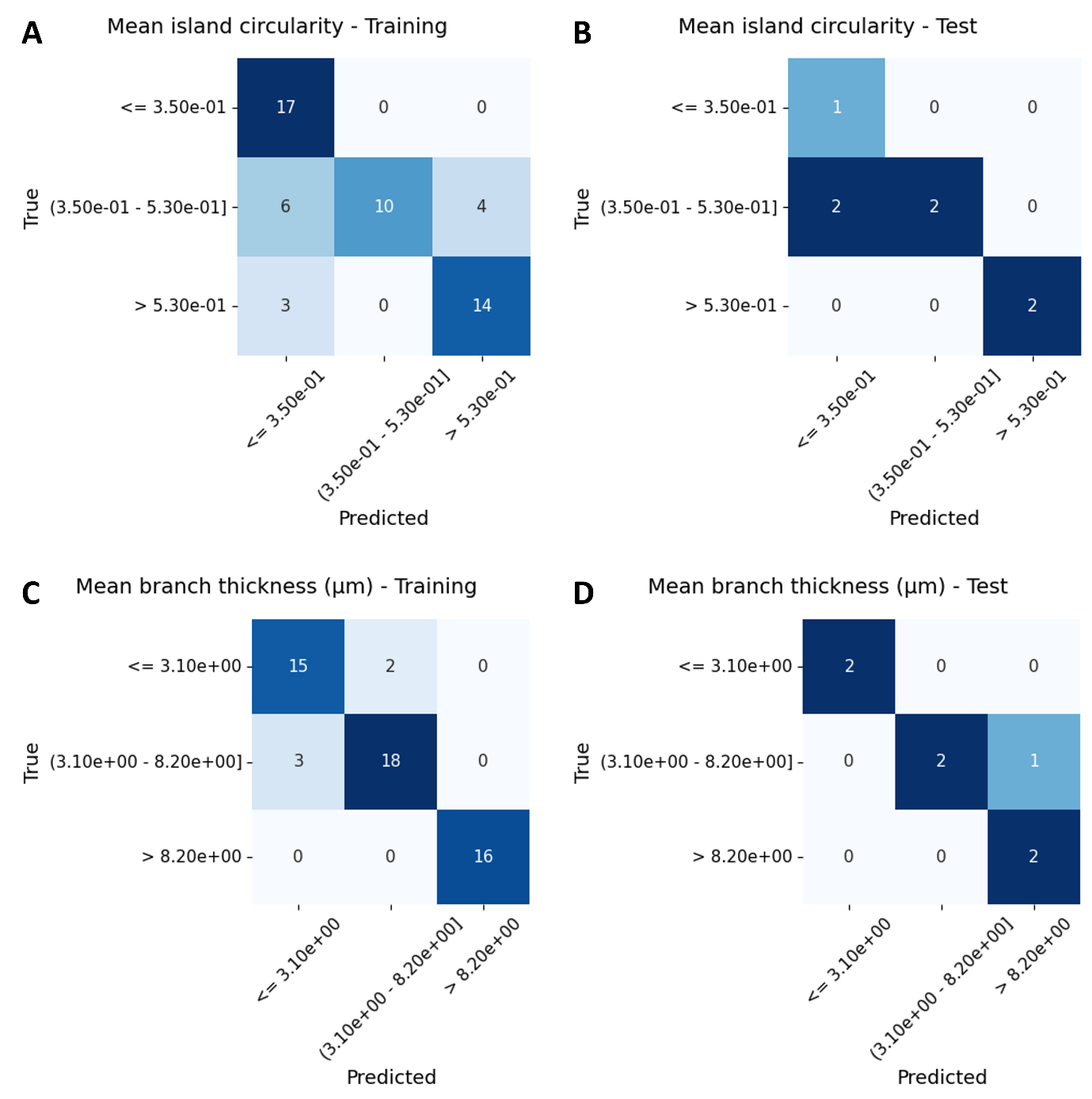


**Figure S4.** Confusion matrices for the ordinal cumulative model predicting (A,B) mean island circularity and (C,D) mean branch thickness (μm). Panels (A,C) represent training results, and (B,D) represent test results.


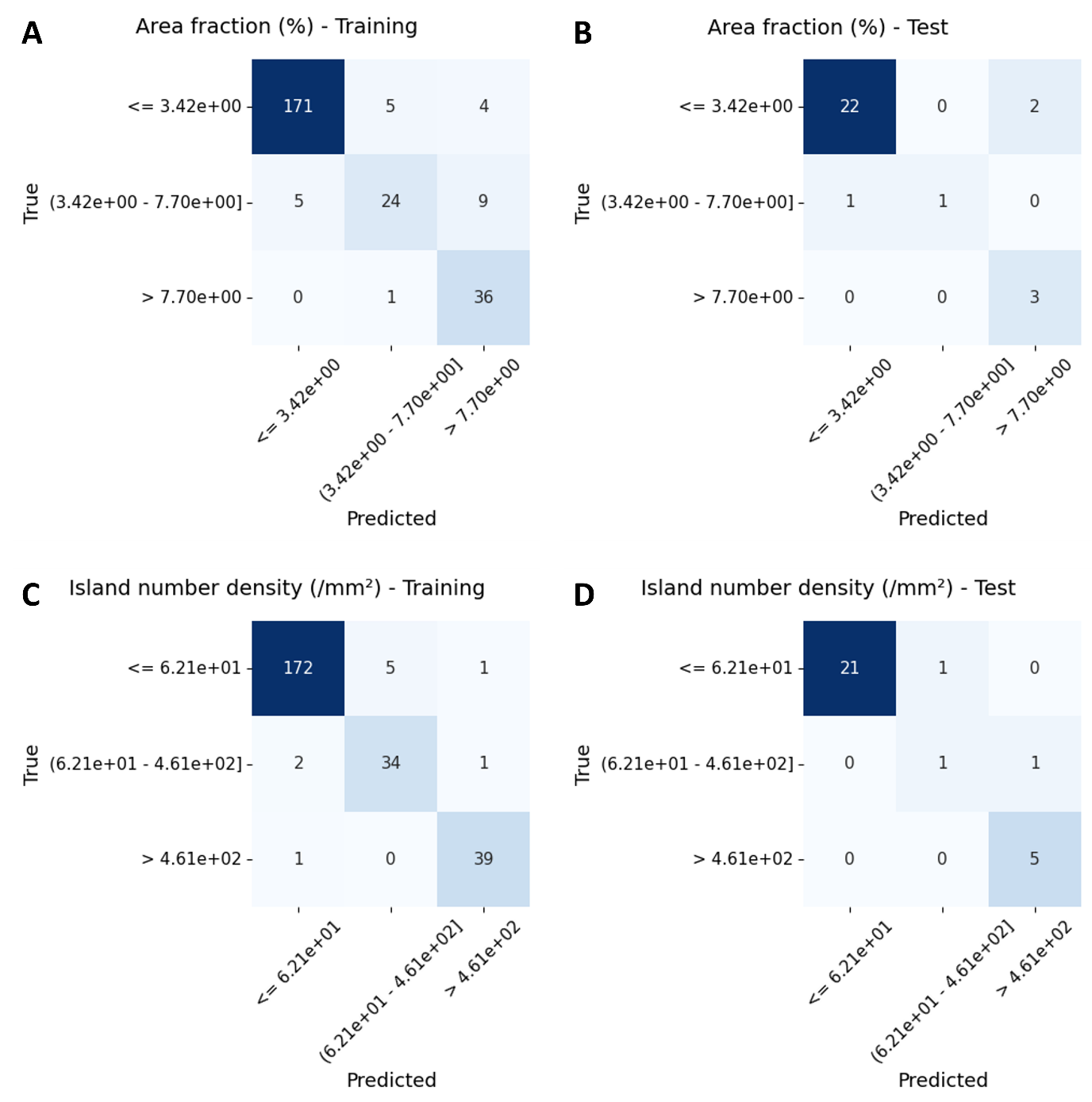


**Figure S5.** Confusion matrices for the ordinal cumulative model predicting (A,B) area fraction (%) and (C,D) island number density (/mm²). Training results appear in (A,C), and test results appear in (B,D).


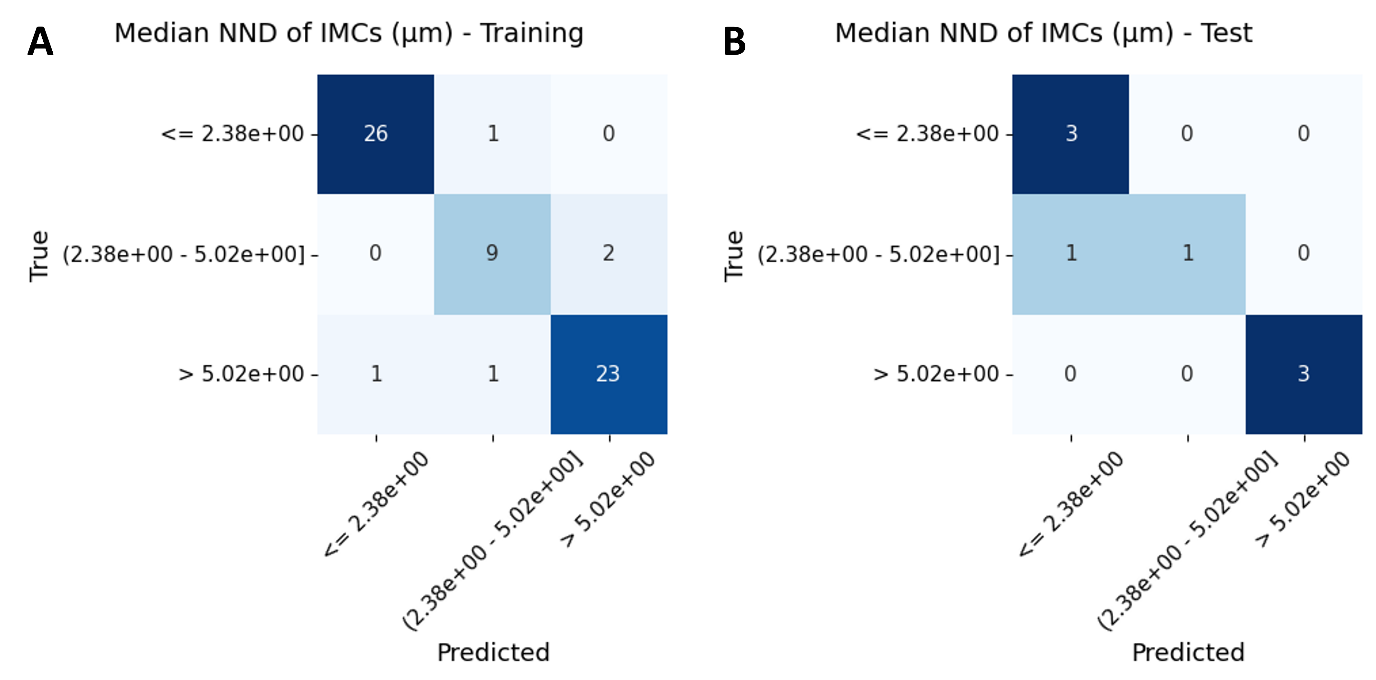


**Figure S6.** Confusion matrices for the ordinal cumulative model predicting the median nearest-neighbor distance (NND) of intermetallic particles (μm). Panel (A) shows the training set performance, and panel (B) shows the test set performance.
